# Supplementary material for: Plasmodium, the Apicomplexa Outlier When It Comes to Protein Synthesis
Source: Biomolecules. 2023 Dec 29;14(1):46. doi: 10.3390/biom14010046 (PMC10813123; doi:10.3390/biom14010046)
Supplement: Supplementary file 1 [file biomolecules-14-00046-s001.zip › biomolecules-2762735-supplementary.pdf]

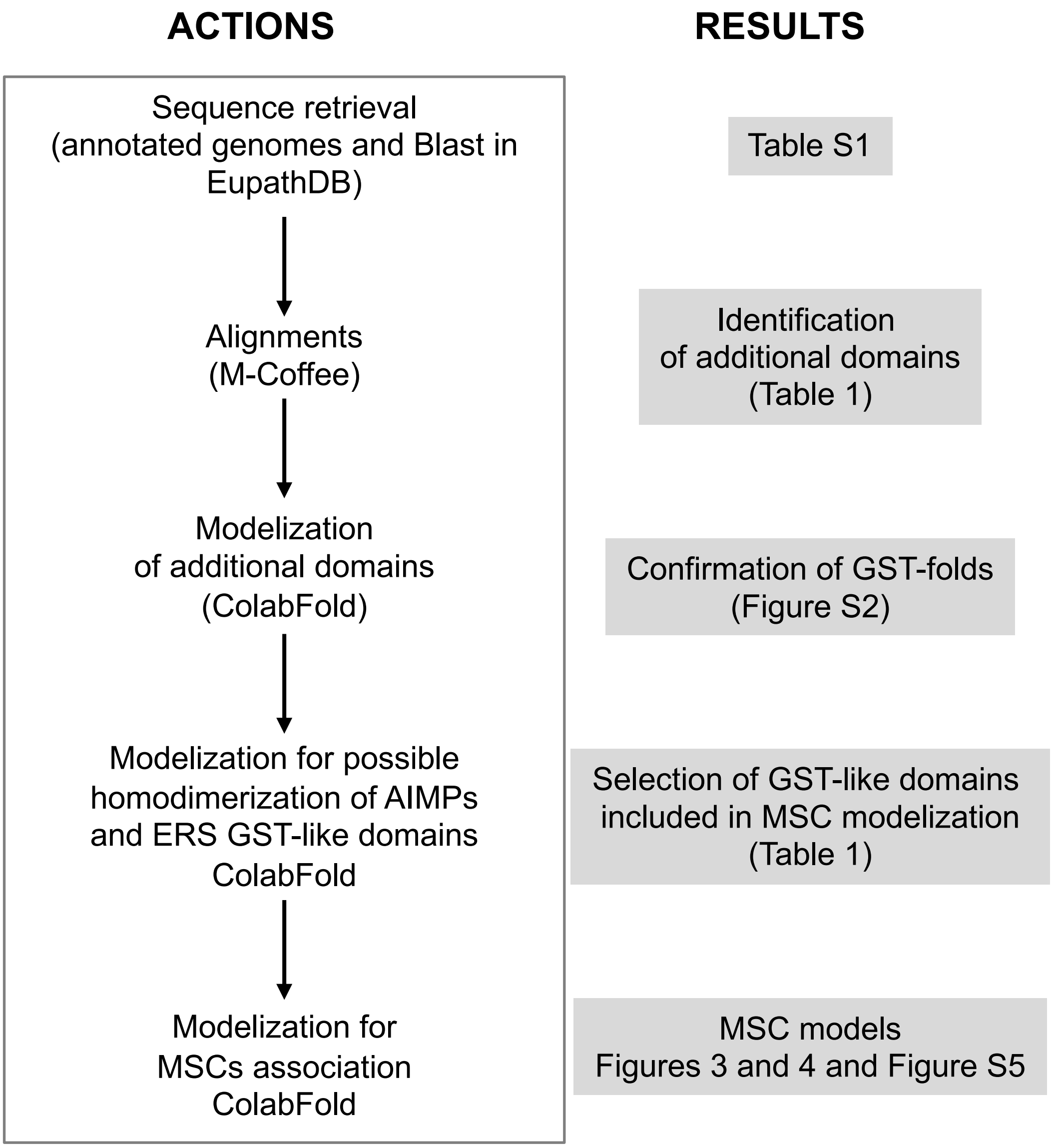

Figure S1: Pipeline for modeling GST-like interactions in MSCs. Global approach used to identify GST-like folds in AIMPs and aaRSs and to model their association into MSCs with ColabFold. The occurrence of canonical interfaces 1, 1’ and 2 in the resulting models suggest a positive interaction between GST-like domains.

Table S1. (A) Annotation of GST-like containing protein genes in EupathDB. (B) Additional domains identified in *Apicomplexa* aaRSs (except GST-like domains)

A

	AIMPs and AARSs					Translation factors			
	AIMP	ERS	MRS	QRS	YRS	EF1-β	EF1-γ	EF1-α	EF1-δ
P. berghei	PBANKA_1306200	PBANKA_1362000	PBANKA_0518700	PBANKA_1346600	PBANKA_1222800	PBANKA_0814200	PBANKA_1352000	PBANKA_1133300	PBANKA_1218900
P. chabaudi	PCHAS_1309400	PCHAS_1366600	PCHAS_0518800	PCHAS_1351200	PCHAS_1223400	PCHAS_0814500	PCHAS_1356600	PCHAS_1133800	PCHAS_1219600
P. falciparum	PF3D7_1442300	PF3D7_1349200	PF3D7_1034900	PF3D7_1331700	PF3D7_0807900	PF3D7_0913200	PF3D7_1338300	PF3D7_1357100	PF3D7_0319600
P. knowlesi	PKNH_1240000	PKNH_1251700	PKNH_0620100	PKNH_1268800	PKNH_0110800	PKNH_0711100	PKNH_1263000	PKNH_1114500	PKNH_0821600
P. vivax	PVP01_1264700	PVP01_1205600	PVP01_0620000	PVP01_1222900	PVP01_0112500	PVP01_0711600	PVP01_1216400	PVP01_1114900	PVP01_0820600
P. yoelii	PY17X_1310000	PY17X_1367700	PY17X_0519800	PY17X_1351500	PY17X_1226000	PY17X_0817500	PY17X_1357200	PY17X_1134900	PY17X_1222100
B. bigemina	BBBOND_0102100	BBBOND_0404120	BBBOND_0207070	BBBOND_0307760	BBBOND_0202210	BBBOND_0110635	BBBOND_0200780	BBBOND_0108320	
B. bovis	BBOV_III010240	BBOV_III011840	BBOV_I001970	BBOV_III006400	BBOV_II002250	BBOV_IV003370	BBOV_II000890	BBOV_IV010620	
B. microti	BMR1_01G02705	BmR1_04g04960	BMR1_02g02000	BMR1_03g03040	BMR1_01G00295	BMR1_03g01425	BMR1_02g03070	BMR1_03g03725	
T. annulata	TA11475	TA17035	TA05325	TA12530	TA08075	TA16305	TA08705	TA06720	
T. orientalis	TOT_020000112	TOT_010001344	TOT_030000712	TOT_020000303	TOT_040000556	TOT_010001036	TOT_040000424	TOT_010000685	
T. parva	TpMuguga_02g00124	TpMuguga_01g01053	TpMuguga_03g00137	TpMuguga_02g00322<br>TpMuguga_02g02375	TpMuguga_04g00367	TpMuguga_01g01096	TpMuguga_04g00492	TpMuguga_01g00726	
C. hominis	Chro.40444	Chro.80098	Chro.80398	Chro.10244	Chro.60344	Chro.20423	Chro.70492	Chro.60459	
C. muris	CMU_018940	CMU_024700	CMU_006170	CMU_028890	CMU_031000	CMU_003010	CMU_014590	?	
C. parvum	cgd4_3880	cgd8_790	cgd8_3460	cgd1_2130	cgd6_2970	cgd2_3950	cgd7_4450	cgd6_3990	
T. gondii	TGME49_223140	TGME49_263870	TGME49_289300	TGME49_217460-t26	TGME49_251880	TGME49_226410	TGME49_300140	TGME49_294800	TGME49_219140
N. caninum	NCLIV_006150	NCLIV_024090	NCLIV_041650	NCLIV_062220	NCLIV_066920	NCLIV_046550	NCLIV_064580	NCLIV_014180	NCLIV_061340

B

		ARS	CRS	DRS	FRS-α	FRS-β	GRS	HRS	IRS	KRS	LRS	NRS	PRS	RRS	SRS	TRS	VRS	YRS	WRS
**Nter**	*Plasmodium*	ASP	ASP	K-rich helix	DNA-binding		ASP	?	K-rich helix	K-rich helix		K-rich helix	Ybak			ASP			Ala-X
	Toxoplasma		?	K-rich helix	DNA-binding		ASP	?	-	K-rich helix		K-rich helix	Ybak			ASP			Ala-X
	Neospora		?	K-rich helix	DNA-binding		ASP	?	-	K-rich helix		K-rich helix	Ybak						Ala-X
	Babesia			K-rich helix	DNA-binding		ASP	?	-	K-rich helix		K-rich helix	Ybak						Ala-X
	Theileria	ASP		K-rich helix	DNA-binding		ASP	?	-	K-rich helix		K-rich helix	Ybak			ASP			Ala-X
	Cryptosporidium			K-rich helix	DNA-binding			?	-	K-rich helix		K-rich helix	Ybak						
**Cter**	*Plasmodium*								Zn binding		tRNA binding		Zn binding						
	Toxoplasma								Zn binding		tRNA binding		Zn binding						
	Neospora								Zn binding		tRNA binding		Zn binding						
	Babesia								Zn binding		tRNA binding		Zn binding						
	Theileria								Zn binding		tRNA binding		Zn binding						
	Cryptosporidium								Zn binding		tRNA binding		Zn binding						

ASP: Apicoplast Signal Peptide
?: Peptide with no fonctional prediction
Apicomplex- specific domain
Eukaryote-specific domain

A EF1β

PLASMODIUM

```
                                                     α5                    α7        α
P.vivax        M-ASNANLLTVKGEGDYGKLNAFFAEHSYYQNHMLSAMDLKIFNQIKCVVSKDTYPHLYRWYKHIDSLPSYVTDQFKD
P.yoelii       MASNNANLLTVKGESDYGKLNSFFAENSYFENYMLSGNDIKIYNQIKCVINKDSYPHLYRWYNHISSLPNYVLDKYMD
P.berghei      MASNNANLLTVKGESDYGKLNSFFAENSYFENYMLSGNDIKIYNQIKCVINKDTYPHLYRWYNHINSLPNYVLDKYMD
P.knowlesi     M-ASNANLLTVKGEGDYAKLNAFFAEHSYYQNHMLSAMDLKIYNQIKCVVCKEKYPHLYRWYKHISSLESYVTEQFKD
P.falciparum   M-ASNASLLNVKGEEDYHNLNSFFESHSYVDNYSVGFKDIKIYHQIKTVIKKDTYPHLFRWFHHISSLPEFILNQYDE
P.chabaudi     MASNHANLLTVKGESDYGKLNSFFAENSYFENYMLSANDIKIYSQIKCVINKDTYPHLYRWYHHISSLPKYVLDKYMD
               * :.:*.**.**** ** :**:** .:** :*: :.  *:**: *** *: *:.****:**::**.** .:: ::: :
```

THEILERIA

```
                                                   α5                 α7
T.annulata     MADLKVDFDSLGKTEGLLNLDKFLSYHSFVGGVKNATTDDVAVFKKLGKAPAEEEYPNVYRWYLHVSTWCNS
T.orientalis   MADVKLNFDLLGKPEGLADLDKYLSYHSFVGGVKNATTDDVAVFNKLADTPSQSAYPNVYRWYLHVKTWHKN
T.parva        MADLKVDFDSLGKAEGLLNLDKFLSYHSFVGGVKNATTDDVAVFKKLGKAPAEKDYPNVYRWYLHVSTWCHS
               ***:*::** ***.*** :***:*********************:**..:*::. ***********.** :.
```

BABESIA

```
                                                                                    α5
B.microti      MSNPEPIVSRPKGWIPMGLATMCSDIIKAIDNDNLKPLDDRLKLCSFISGPLKPNEKDLEIFKMLKKAP-AGKFLHACRW
B.bigemina     M------------------PDFKVDLSKVASREGLVELNEYLADHSFVAGCKAPSTDDLAVFRKLGNSPGAAEFAHVDRW
B.bovis        M------------------ASVKIDINTVSSASGLSQLNEFLSYHSFVGGVKAPSTDDIAVYKKLGSAPDANAYPHVARW
               *                  . .  *: .. . ..*  *:: *   **:.*   *. .*: ::: * .:* *  : *. **

               α7
B.microti      YNHIDALVKK
B.bigemina     FRHVSTWAQN
B.bovis        YKHVSTWVQN
               :.*:.: .::
```

CRYPTOSPORIDIUM

```
                                                 α5                α7
C.hominis      MDK---FGDISTDAGRKKLNDYLSTRSYMNGVSPTQDDVEALYMIIKAGG-FCDFQHICRYVSHIQS
C.parvum       MDK---FGDISTDAGRKKLNDYLSTRSYMNGVSPTQDDVEALYMIMKAGG-FCDFQHICRYVSHIQS
C.muris        MLKIKGFGDLSTLEGKKKLDEFLSNRTYFNGVNPSQNDCNLVSELLLAGTKYCSFPHICRYLEHMRH
               * *   ***:**  *:***::**.*:*:***.*:*:* : :  :: **  :*.* *****:.*::
```

TOXOPLASMA/NEOSPORA

```
                                              α5                α7
T.gondii       MVVPDFGNLKSDIGVGKLNEYLASRSFITGYSATQDDAIIFSKLLGAPNATKFVDASRWYRHVSHFSPVQKAAW
N.caninum      MVVPDFGNLKSDIGVGKLNEYLASRSFITGYTATQDDAIIFSKLLGAPNATKFVDASRWYRHVSHFSPVQKAAW
               ******************************:*******************************************
```

B EF1γ

PLASMODIUM

```
                 β1        α1                                   β2    β3    α2
P.vivax        MDFKLLAPKNDIRSLKVQVVASFCNVKLNIPQFEIGKDDKTQEFLNYSPLGRLPVLVTSSGSLFESNAISKYLCNIRREN
P.yoelii       MDLKLLGPKNDIRCLKVQTVASFCNIKLNMPAFEIGIDDNKDEFVKESPMKRLPVLITPQGSLFESNAIGKYLCSIRKEH
P.berghei      MDLKLLGPKNDIRCLKVQTVASFCNIKLNIPTFEIGIDDNKDEFIKESPMKRLPVLITPQGSLFESNAIGKYLCSIRSEH
P.knowlesi     MDFKLLAPKNDIRSLKVQAVASFCNVKLNIPNFEIGKDDKTQEFLDYSPLGRLPVLVTPSGSIFESNAISKYLCNIRREN
P.falciparum   MDFKLLAPKNDVRTLKVQTVASFCNVKLNMPNFELGKDNKTADFLKHSPLGRLPVLVTSHGSIFESNAVCKYLCSIHREG
P.chabaudi     MDLKLLGPKNDIRCLKVQTVASFCNVKLNMPAFEIGVDDKKDEFVKESPMKRLPVLITPDGSLFESNAIAKYLCSIRREH
               **:***.****:* ****.******:***:* **:* *:. :*:. **: *****:*. **:*****: ****.*: *

                            α3                        α4                     α5
P.vivax        NLLGKGAFEEAQVNMWVDFNTYELEIPVCCYISNKSNEKSLKHIQETFACLNNHLLLNQFMVGNGITLADIFICVIINFA
P.yoelii       NLLGCGCFEEGQVNMWIDFCTFELEIPVCCYISNKSNDKSLKHIQDTFSCLNKHLLLNQYMVGNKITIVDIFMSVIINFC
P.berghei      NLLGNGIFEEGQVNMWVDFCTFELEIPVCCYISNKLNEKSLKHIQDTFSCLNKHLLLNQYMVGNNITIVDIFMSVIINFC
P.knowlesi     NLLGKGVFEEAQVNMWVDFNTYELEIPVCCYISNKSNEKSLKHIEETFACLNNHLLLNQFMVGNGITLADIFICVIINFA
P.falciparum   DYLGKGSFEEAQVNMWVDFNTYELEIPMTCYMNNKSCEKSLKHIEDTLKCYNNHLLYNQYMVGNSITIADIFISVILYFS
P.chabaudi     NLLGKGSFEEGQVNMWVDFCTFELEIPVCCYISNKSNEKSLKHIQDTFTCLNKHLLLHQFMVGNNITLVDIFMSVIINFC
               : ** * ***.*****:** *:*****: **:.**  :******::*: * *:*** :*:****.**:.***:.**: *.

                  α6     α7      α8
P.vivax        MKSEKMDEGFLKKYVNLFRLYNTISNQKQFKYVFASQ
P.yoelii       IKSGKMTEDFLKKYGNLYRLYTTIINQKQFKYVMGSG
P.berghei      IKSGKMTEAFLKQYGNLYRLYTTIINQKQFKYVMGSG
P.knowlesi     MKSEKINENFLKKYGNLFRLYNTISNQKQFKYVFASQ
P.falciparum   LNSGALNESIVLKYKNLYRLYDTISNQKQFKYVFACD
P.chabaudi     IKSGKMTEDFLKKYGNLYRLYTTIINQKQFKYVMGSG
               ::*  : * :: :* **:*** ** ********:..
```

Figure S2

THEILERIA

```
                    β1          α1           β2                    β3     β4     α2
T.annulata     MVRKVVGVDLEDYGTKSVLVTAAWANVNFDFEVAKGTCCASQAKKNAFSELKHAVTLVLDKDLGTFCGHVTVCRHLLDHV
T.orientalis   --MKVVGVDVDDYGTKCVLVTAAWSDLKFDFEASKGVCCSSTCEKDAFSELKHAVTLVQDNDLGTFCGHVSVCRHLMDLV
T.parva        --MKVVGVDVDDYGTKSVLVTAAWANLNFDFEVAKGTCCASQAKKNAFSELKHAVTLVLDKDLGTFCGHVTVVRHLLDQV
                 ******::*****.*******::::****.:**.**:*  .:*:************ *:*********:* ***:* *

                             α3                              α4                       α5
T.annulata     SKGTDEKSAFERSDTSSWVEFFYSRLDFETKLDKSSVVTRGSNSDLSKVVVSLNNYLATKTFLVAEKLSPADLVCAVSLD
T.orientalis   CRGTDDKAVFVKGDTSSWLEFFYLRLEMESKLDTKSVVTKGDNSDLSKVVHAMNKYLATATFLVCEKLGPADLVFAVTLE
T.parva        SKGTDEKSAFERSDTSSWVEFFYSRLDFETKLDKSSVVTKGSNSDLSKVVNSLNNYLATKTFLVAEKLSPADLVCAVSLD
               .:***:*:.* :.*****:**** **::*:***..****:*.******** ::*:**** ****.***.***** **:*:

                         α6     α7             α8
T.annulata     HLVHQKRLDAEYLEKELLHLARFLRTVKASDSFQKFLKMVENYKST
T.orientalis   HLMHHKRLDAAYLEAELLHVARFLRTVKASDKYQKFLEMVERFRGA
T.parva        HLVHQKRLDPEYLEKELLHLARFLRTVKASEAYQKFLKMVENYKSN
               **:*:****. *** ****:**********: :****:***.::.
```

BABESIA

```
                                β1          α1           β2                    β3
B.microti      -----------------MQIIGD-NNSVQTKIAVALTVFSGH--DPKVLIGNGNETSLLPQSF-KLMPMLKEST-----N
B.bigemina     MVGCASAAAALTSLTHLQKVIGVDFNDLGTKNVLAAAQFLGVAYDFQLSKGSCCSSEGKKDEFSEVKPCVSVELCGGLGA
B.bovis        -----------------MKIIGVDLTDLGTKNVLAAAAFYNVSYDFTLAKGGCCSSEGKKDEFSDVKPSVSVELSGGLGS
                                 ::**   ..: ** .:* : * .    *  :  *.  .:.   :.* .: * :. .

               β4   α2                      α3                                  α4
B.microti      HVGTLTIYNYLSGKYAKNFFGATTETRNQITTLFDY------------SVTNLMKYLHLPLGDCLKNKLSELNKCFLKNT
B.bigemina     FCGSISICRHMMEQKKKGDSPAELFARSDATSWMEFFYDRFDVGSRCQAVTQVDASSTSELAKCLR----ATDAFLLSRT
B.bovis        FCGSISISRHLMEEHKKGSAPEELFARSDITSWMEFFYERLDVGSRCQAVTKVVHGDHSELAKCIR----AIDAFLLTHT
               . *:::* .::  :  *.         :*.: *: :::           :**::        *..*::       :  :*..*

                         α5                 α6      α7           α8
B.microti      FFVEDQITAADVSMAIIIEELLSRNIIDNCIMENDYYCVKRWFETVTAQDLYKKY---LCTLKGASS
B.bigemina     FLVGESLSVADIVCAVTVKHLVSIGKLEAGYLRTGLRNLSRLIRTVEASDAFVNYEKKLASLQSGSA
B.bovis        FLVGEALSLADVVCAITVDHLISISKLDTEYLASRLLNLSRLIRTVKESDVYAAYHKKLATLNSGVA
               *:*  : :: **:  *: :..*:* . ::    : .     :.* :.**  .* :   *    *.:*:..
```

CRYPTOSPORIDIUM

```
                    β1          α1          β2                     β3     β4     α2
C.hominis      --MVYTLFGLDSCPLVSIIKSVAEITGDKISHKVSDGTDHKDLDFHLTF-GTPVLDTQFGKIFGTSTILRFLARGSPYTN
C.parvum       --MVYTLFGLDSCPLVSIIKSVAEITGDKISHKVSDGTDHKDLDFHLAF-GTPVLDTQFGKIFGTSTILRFLARGSPYTN
C.muris        MNTEHKVYVMKKCPLSVVIASVAEIAQVKVKVIDRNEFTEERQSSLIFGDKTPVISTPFGDIFGIHSILRYLARCAPASF
                   :.:: :..***  :* *****:   *:.     :    .:   .   :     ***:.* **.***   :***:*** :* :

                            α3                              α4                        α5
C.hominis      LYGRTDSDATHVDMILQYIDTTLSSLVVPLFKKSGD--VKSYVSE--LTKIFESFNKYFNYRTFVAGERITIADILLATI
C.parvum       LYGRTDSDATHVDMILQYIDTTLSSLVVPLFKKSGD--VKSYGSE--LTKIFESFNKYFNYRTFVAGERITIADVLLATI
C.muris        LYGETLYESALVDALLEGFHSNILKPIKALCSTACPSECKSDKSEVDLGTVLSNLDDYLLSRTFLAGHRVTIADILIAVV
               ***.*   ::: ** :*: :.:.: . : .* ..:        **   **   *  .::..:::.*:  ***:**.*:****:*:*.:

                                 α7           α8
C.hominis      IDYSIKCSAGALSLKSCVNLQRYVDTILAQPSIKKHYGEYVKKCSASKDSQAK
C.parvum       IDCSIKCSAGALSLKSCVNLQRYIDTILAQPSIKKHYGEYVKKCSASKDSQAK
C.muris        ICFTVDISKGSLSINGLVNLSRFVNTIMNNYIIRKHYD-NLKKYQTCTDSNTN
               *  ::.  * *:**::. ***.*:::**: :  *:***.  :**  .:..**:::
```

TOXOPLASMA/NEOSPORA

```
                  β1       α1          β2                       β3     β4     α2
T.gondii       MKLLTPKDDVRGRKVQLVAAFLDLPLQTVPFTVGKDDKDPAFLAKSPLGRLPLLESEVGGVCLFESNAICRFLARLRADK
N.caninum      MKLLTPKDDVRGRKVQLVAAFLDVPLQTVPFTVGKDDKDPAFLAKSPLGRLPLLESEVGGVCLFESNAICRFLARLRADK
               **********************:*********************************************************

                              α3                             α4                         α5
T.gondii       CLYGETLAEQGQVDMWLDFSTLEVEIPMCCLVQGGKVAERAQSDLAQALNAVDAHLKTRTFMVGENITIADLCLVAVLSY
N.caninum      CLYGETLAEQGEVDMWLDFSTLEVEIPMCCLVQGGKVAERAQSDLSQALNAVNAHLKTRTFMVGENITIADLCLVTVLSY
               ***********:*********************************:******:**********************:****

                          α6      α7        α8
T.gondii       GFRSGKVDAAALLEKRPYLKRFYETVVNQKSFKKIFGE
N.caninum      GFRSGKVDCAAILGQRPFLKRYYETLVNQKSFKKVFGE
               ********.**:* :**:***:***:********:***
```

Figure S2

C AIMPs

PLASMODIUM (tRip)

```
               β1        α1                      β2        β3    β4    α2
P.vivax        MCVLTLVQDDVKSDILKLVLDFIKAVVVKDDEKVAFPEVRHEKKISFQYKDKQYKELFCTLYAIIDIYDCYNELFNEDEG
P.yoelii       MCTLNLVEDDIKSDILKLALDFIKTNIVENDDNVVFPEIKYGQKISYEHNNKTYKEFFCSLYAIIDTYNCYSQFFCEDED
P.berghei      MCTLNLVENDIKSDILKLALDFIKTNIVENDDNVVFPEIKYGQKISYEHNKKTYKEFFCSLYAIIDTYNCYSQFFCENED
P.knowlesi     MCVLTLVQDDVKSDILKLVLDFIKAVVIKDNEKVAFPEVRHEKKISFEYKEKQYKELFCTLYAIIDIYNCYNELFNEDEG
P.falciparum   MCVLTLVKDDIKSDILKLVLDYIKVTVVQDNENVKLPEICYDKKITLQYKNKTYKDLFCTLYALIDIYDCYSELFNEDEG
P.chabaudi     MSTLYLVEDDIKSDILNLVLDFIKINIVENDDSVAFPEIKYSQTISYEHNNKTYKEFFCSLYAIIDTYNCYSQFFCEDEN
               *..* **::*:*****:*.**:**  ::::::.* :**: : :.*: :::.* **::**:***:** *:**.::* *:*.

                  α3               α4                   α5              α6      α7
P.vivax        KVSENEEFIFHLASDKFKLKQLDMKHLNDLLCEKSYIVSNRHASIVDIFYFCSVYKPLSEMPAKERVEISHIYRWFLHIQ
P.yoelii       KVSESEEFIFNLASDKFKLKPLDMKHLNDILRERSYIVSDKHASIVDIFYFCCVYKILKPMTAKERVEYYHICRWYIHLQ
P.berghei      KVSESEEFIFNLASDKFKLKPLDMKHLNDILRERSYIVSDKHASLVDIFYFCCVYKILKPMTAKEKVEYYHICRWYIHLQ
P.knowlesi     KVSENEEFIFHLASDKFTLKQLDMKHLNDLLCEKSYIVSNRHASIVDIFYFCSVYKLLSEMPPKERVEFSHIYRWFLHIQ
P.falciparum   KVSENEEFIFHLASDKYILKQSDMKHLNDLLCEKSYIISNKHASIVDIFYFCAIHKLLDEMAVKERIEFSYIYRWYLHIQ
P.chabaudi     KVSESEEFIFNLASDKFKLKPLDMKHLNDILRERSYIVSDKHASIVDIFYFCCVYKILKPMAAKEKVEYYHICRWYIHLQ
               ****.*****:*****: **  *******:* *:***:*::***:*******.::* *. *. **::*  :* **::*:*

                          α8
P.vivax        ETLVGKFTTLKKLEVRDSLETFLNSKNV---------------MSPGERANSVSMRQQKDDKNAKNENGDNEKGKKKNNA
P.yoelii       ETLICDFVKLHKLDIRSGVESLLNNRTV---------------TCTDEKVSNEQVSPKKDKPNKKNADSK--DNN-GKNK
P.berghei      ETLICDFVKLHKLDIRSGVESLLNSGIV---------------TCTNEKASNEQVGSKIDKANKKNSDSK--DNN-GKNK
P.knowlesi     ETLVGNFTTLKKLEVRDSLETFLNSKNVVNPSERANNAGAGQQKDDKNVKNANGENAENGQNGQNVQNVQNEKSKKKNNA
P.falciparum   ETLLANFSTLKKLIVKDSLENLLNNKTT----------------NNAPEHKNNFVSKESKENKSQNNESPK--NK-KKDV
P.chabaudi     ETLMCEFVKLHKLDIQSGVESLLNSRII----------------TSTNEKGNNEQMGSKKDKGNKKNADSK-DNS-GKNK
               ***: .* .*:** ::..:*.:**.

P.vivax        QNKNAQ-KKKVEEPKNLDDITRLNIIVGYVEEVEIHPDADTLYCLKINVGEEKSRDICSGLRLKKNSEDLLHKYVLVLAN
P.yoelii       KKNNNAENKDAEESRSLDDISRLNIVVGYVESVEIHSGADTLYCLKVNVGEDQLRDICSGLRNKKNPEDLLNKYVLVLAN
P.berghei      KKSNNAENKDAEDSRSLDDISRLNIVVGYVESVEIHSGADTLYCLKVNVGENQLRDICSGLRNKKNPEDLLNKYVLVLAN
P.knowlesi     QNKNNQ-KKKVEEPKSLDDITRLNIIVGYVEEVEIHPDADTLYCLKINVGEEKSRDICSGLRLKKNSEDLLHKYVLVLAN
P.falciparum   QNKNNAPNKKVEETKKLDDISRLNVLVGYVEQVEIHPDADTLYCLKINLGEDKPRDICSGLRNKKNAEDLLNKYVLVLAN
P.chabaudi     KKNNNAENKDAEETRSLDDISRLNIVVGYVESVEIHSGADTLYCLKVNVGEDQVRDICSGLRNKKNPEDLLNKYVLVLAN
               ::.*   :*..*:.:.****:***::*****.****..********:*:**:: ******** ***.****:********

P.vivax        LKEKSLRGRKSHGMVLCGSFGEQIELLAPPDGVNVGERIICENMDVNKLPDKTLSFDKEKNPFFHIQPHLLVKNGVAHYK
P.yoelii       LKEKSLRGRKSFGMILCGSFEERVELLTPPPGVKIGERITFENMNTNSLPDKTLSSVKEKNAFFLIQPNLLINNGVAFYK
P.berghei      LKEKSLRGRKSFGMILCGSFEERVELLTPPPGVKIGERITFENMNTNSLPDKTLSSVKEKNAFFLIQPNLVINNGVAFYK
P.knowlesi     LKEKSLRGRKSHGMVLCASFGEQIELLAPPSGVKVGERIICENMDVNKLPDKTLSSDKDKNPFFHIQPHLLVKNGVAYYK
P.falciparum   LKEKSLRGKKSHGMVLCGSFDEKVELLVPPNGVKIGERILFHNMDPNVIPDKNLSSDKEKNPFFHIQPHLILKDGVAHYK
P.chabaudi     LKEKLLRGRKSFGMVLCGSFEERVELLVPPPGVKVGERITFENVNTTGLPDKTLSSVKEKNAFFLIQPNFVINNGVAFYK
               **** ***:**.**:**.** *::***.** **::****  .*:: . :***.**  *:**.** ***:::::***.**

P.vivax        DAKWLSSKGEITCPLEQGTIS
P.yoelii       NNKWLSSQGEITCAIEQGTIS
P.berghei      NNKWLSSQGEITCMIEHGTIS
P.knowlesi     DTKWLSSKGEIICPLEEGTIS
P.falciparum   DTKWISSQGDITCVLNQGTIS
P.chabaudi     ENKWLSSQGEITCALDQGTIS
               : **:**:*:* * ::.****
```

THEILERIA (*Tp*-p42)

```
               β1         α1          β2            β3                α2
T.annulata     MLELTVNSNSQESQISKLLWKYLNLNHATLLFTTDKDTESALKFKDGRETVKHFPLILELVLTTSDTGSLLIPNEPELKG
T.orientalis   MIELVVYSDSQESQIVKLLLKYLNLNDVSLTLSTNKDEELFLKLPGETEPVKHFPLMLERLLKSSPHGSLLLPTDPELKA
T.parva        MLELTVNTNSQESQISKLLWKYLNLNHATLLFTTDNDTESALKFKDGRETVKHFPLILELVLTTSDTGSLLIPNEPELKG
               *:**.* ::****** *** ******..:* ::*::* *  **: .  *.******:** :*.:*  ****:*.:****.

                 α3                α4                   α5           α6      α7
T.annulata     TMDSFVDFAFKHNFDVLDVESIRMLDTYLLNETFFAGPTLTLADVVLFVSLLNWTVRSKPKERSEFPNLMRWFDHLQHLP
T.orientalis   TMDSFVDFGFKHGFNVLDVDSLRMLDNYLLNETFFSGPTISLADVVLFVSVTYWTSRSKSKERMEVPNLMRWFDHLQHLP
T.parva        TMDSFVDFAFKHNFDVLDVESIRMLDTYLLNETFFAGPSLTLADVVLFVSVLNWTVRSKPKERSEFPNLMRWFDHLQHLP
               ********.***.*:****:*:****.********:**:::*********:  ** ***.*** *.*************

T.annulata     ELATCFEEFSVINLFDVCGIKLDFI-FRTNSLYRSLRRKKLKRKKKRKK--------NRSFEG----------------
T.orientalis   EFASCFEEFKVIQLFDEKLVMTEPKKGKPRAAYHPSEKAEKKKEKKEKPKVEKPPVETRPYDDVTRLNVVVGLVKSIRKH
T.parva        ELAACFEEFSVINLFDNKLVISQPK--AEKVE--------KKKEKKEKPKIQAPPAETRPYDDVTRLNVVVGSVLSIRKH
               *:*:*****.**:***   :  :      .

T.annulata     ---CRWQCIVNQEAPGCRQSICSGLVDFLAPDDILNKKVCVLANLPKKNLRGEESNGMVLCVSNEDKTSVELLEPPTETP
T.orientalis   EDADKLYCLKID-VGSEVRSICSGLVDFLMPDQILDKKVCVLANLPKKNLRGEESNGMVLCVSNSDKSSVELLEPPVDAP
T.parva        QDADKLYCLKVD-IGTEVRSICSGLVDFLGPDDILNKKVCILANLPKKNLRGEESNGMVLCVSNEGKSSVELLEPPEETP
                  :  *:  :      :********** **:**:****:**********************..*:******** ::*

T.annulata     VGERVNWEGLSGEPDEQLSLKKGKDTFSLVQKDLLCKDNIGYYKVDLFFFKKSF-------SHRNS
T.orientalis   VGERVYWEGYSGEADEQLSSKKGKDTFAMVQKDLNCKENVGFYKVSGA-----------------E
T.parva        VGERVSWEGLSGEPDEQLSLKKGKDTFAMVQKDLVCKDNVGYYKSSKFMTSKGPCFCKKISTGTIS
               ***** *** ***.***** *******::***** **:*:*:**  .                  .
```

Figure S2

BABESIA (*Bb*-p43)

```
              β1        α1               β2         β3   β4     α2
B.micro ti    MIPLEVHKASNYANIVKLLLSYSNIKDDLIKLTLSDNPQLTLTLPNGTQYTEL-PTIMIYIMGQNDTGKILYPDDNLLK-
B.bigemina    MLEVVVYKDCVEGRLATLLWGYGKLTSA--TLSFAPSRTGMVVKEDGKETPMIWPTLLELAVKDAPTESILVPATEAQKV
B.bovis       MLEIVVYSDCVEGRLARLLWGYGKFNNA--TLSLSSTRTETLVKENGEQSLKQWPDIIEIAVKGASTEDVLVPPTPDQK-
              *: : *:. .  ..:. ** .*.::..    *::: .    :.  :* :      * ::    :     * .:* *      *

                         α3                    α4                         α5            α6
B.microti     ------------AKIDQWISLANKKDYSICDSESIREIDSSLVHTTFLVCNNITLADIVIYSSLLHWAKKSTTKEKELMC
B.bigemina    SALRPVFNFACQRTMYSWIDFAYQRDFSIMESASLKILNDYILNHTFLAGPTVTLADLVVYVSTHSWMMKSEPQDRAEYV
B.bovis       ------------NKMYSWIDFAFQRNFMINQKDSLKILNVYLKPNTFLVGNNVTLADLVAYVSIHSWMLMSEPHDRIEFS
                          .: .**.:* :::: * :. *:: ::  :    ***.  .:*****:* * *    *    * .:::

                 α7                      α8
B.microti     NLSRWYNHIQHLPGISIGNYIDIP---TGTTVSSLSSMLNNVNVCGDN-KKSNKSAQKAEGKYNKKIQNSNFSNDARPVD
B.bigemina    NVVRWFDHIQHLPGIV-NTFKELPLVSVDKDMDVVTAVMEKATVSAAAPAPAGKQAKGQKGAASKKPKDPKPPADDRPIA
B.bovis       NIVRWFDHIQHLPGIV-NTFKDLPLVVVDKDMDLVTAVMDNVTVTAPS---AKNPTKGAIDAKSKKEKSPKPVVDERPIA
              *: **::********  ..: ::*   ... :. :::::::..*  .      : : ::    .   .** :..:    * **:

B.microti     DITRLAVRVGLVKEISKHPDADKIYCLKIDIAESKLRDICSGLVEHLKPEEIINKKVCVLSNLKPRKIRGVDSNGMVLCV
B.bigemina    DVSRLNIKVGQIMSVERHPEADKLYCLKVDLGEPELRDICSGLVGYLEPEQIMSQYVCVLSNMKPKSLRGKVSNGMILCV
B.bovis       DVSRLNIRVGQVMSVDRHPEADRLYCLKIDLGEPELRDICSGLVGYLQPDEIMSKYVCVLSNMKPKNLRGRVSNGMVLCV
              *::** ::** : .:.:**:**::****:*:.*.:********* :*:*::*:.: ******:**:.:**  ****:***

B.microti     SYNDK--VELLEPPSDSMVGELITWGDLKGEPDVVLSGKSGKNPFEAVQKDLVCKNKIGYYKDLPFSTLSGVCSCNTLIK
B.bigemina    SGPGKTELELLHPAEGAPVGERIIIEDCVGEPDAVLSTKTGKDPFVAVQPEFNCKDTVAYYKDHRFMTSCGPCRCNSLKA
B.bovis       SNADHTQIELLHPADGTPIGERVTIEGFDGEPDAVLSTKTGKDPFVAVQPDFNCKDTIAYYKDHRFMTTLGPCHCNSFTS
              *  .:  :***.*...: :** :    .  ****.*** *:**:** *** :: **:.:.****  * *  * * **::

B.microti     GTIS
B.bigemina    GTIS
B.bovis       GTIS
              ****
```

CRYPTOSPORIDIUM

```
               β1        α1              β2            β3              α2
C.hominis     MAILVKYSKTSNIGKVVSLLVNYLLKQNTELKIEESIIDENSTNEKNINQVVLIKDKENCNDQLENIVDILIYLGSFGKN
C.parvum      MVILVKYSKTSNIGKVVSLLVNYLLKQNTELKIEESIIDENSTNEKNINQVVLIKDKENCNDQLESIVDILIYLGSFGKN
C.muris       MTIKLIINPETSFGTLLILVSNYIINQ----KI--SIGTEYS------NQIASTLILESEGIKLINSVDILKHLGSIYPK
              *.* :  .  :.:*.:: *: **:::*             *      **:.      *. . :* . **** :***:  :

                   α3                  α4                  α5
C.hominis     LLGKDSSLINESKEIIEKLIKINFNFSKGTEMSEFNEMIKGKSFFLGRSLTIVDLVIYISLYSYFESLKDQNQEYIPEYP
C.parvum      LLGKDSSLINESKEIIEKLIKINFNFSKGTEMSEFNEMIKGKSFFLGRSLTIVDLVIYISLYSYFESLKDQNQDYIPEYP
C.muris       IFKQDMEPVMT-K-FLELLSRDSFNTESDDILQHLEDQISGKSFFFGRFLSLLDLVVYSSIHSYFKSMNNMDSTYTPKYP
              :: :* . :   * ::* * : .** ... :..::: *.*****:** *:::***:* *::***:*:: :. * *:**

                α7                 α8
C.hominis     NLSTFFEQIQIVTLIRQSCPEGINYLDLPFFKKKLVKKEKKNTGEHGNNSNHNKQ-ERPLDDPTRIALRVGRILSVERHP
C.parvum      NLSTFFEQIQIVTLIRQSCPEGINYLDLPFFKKKLVKKEKKNTGEHGNNSNNNKQEERPLDDPTRIALRVGRILSVEKHP
C.muris       SLSKFFEFVQIMTPIRQACPENLEYLDLPFFTKKLSNKTKK-----LDNT----SDERSLDDPSRIELRVGKILSVERHP
              .**.*** :**:* ***:***.::*******.*** :* **      :*:    . **.****:** ****:*****:**

C.hominis     TADKLYLEKIDVGEDEPRTILSGLVGVYDLTQKINELVVIVSNLKPRAMRGITSNGMLLCASSAPSGDN--NNSQNQSSK
C.parvum      TADKLYLEKIDVGEDEPRTILSGLVGVYDLTQKINKLVVIVSNLKPRAMRGITSNGMLLCASSAPSGDNNNNNSQNQSSK
C.muris       SADKLYVEKIDIGEEEPRTILSGLVGIYDLTLKINELVIVVANLKPKTMRGIISHGMLLCASLD--GD---------TK
              :*****:****:**:***********:**** ***:**::*:****::**** *:*******    **         :*

C.hominis     EYCEPVSVPKDAKVGELIFYNDFKGEPDVVLNTKTGKDPFAAVQPNYNVSSDLLCRFKESTMMTSAGPVFVENKNLIHGK
C.parvum      EYCEPVSVPKDAKVGELIFYNDFKGEPDVVLNTKTGKDPFAAVQPNYNVSSDLLCRFKESTMMTSAGPVFVENKNLIHGK
C.muris       -LCEPIIVPNGAKVGELITYEGFSGSPDPVLSSKAGKDAFSTVQAHYNISNNSICRYKDIIMMSSAGPLYV-NRIFAGAK
                ***: **:.******* *:.*.*.** **.:*:***.*.:**.:**:*.:.:**:*: **:****::* *: :  .*

C.hominis     LS
C.parvum      LS
C.muris       LC
              *.
```

Figure S2

TOXOPLASMA (*Tg*-p43) / ***NEOSPORA*** (*Nc*-p47)

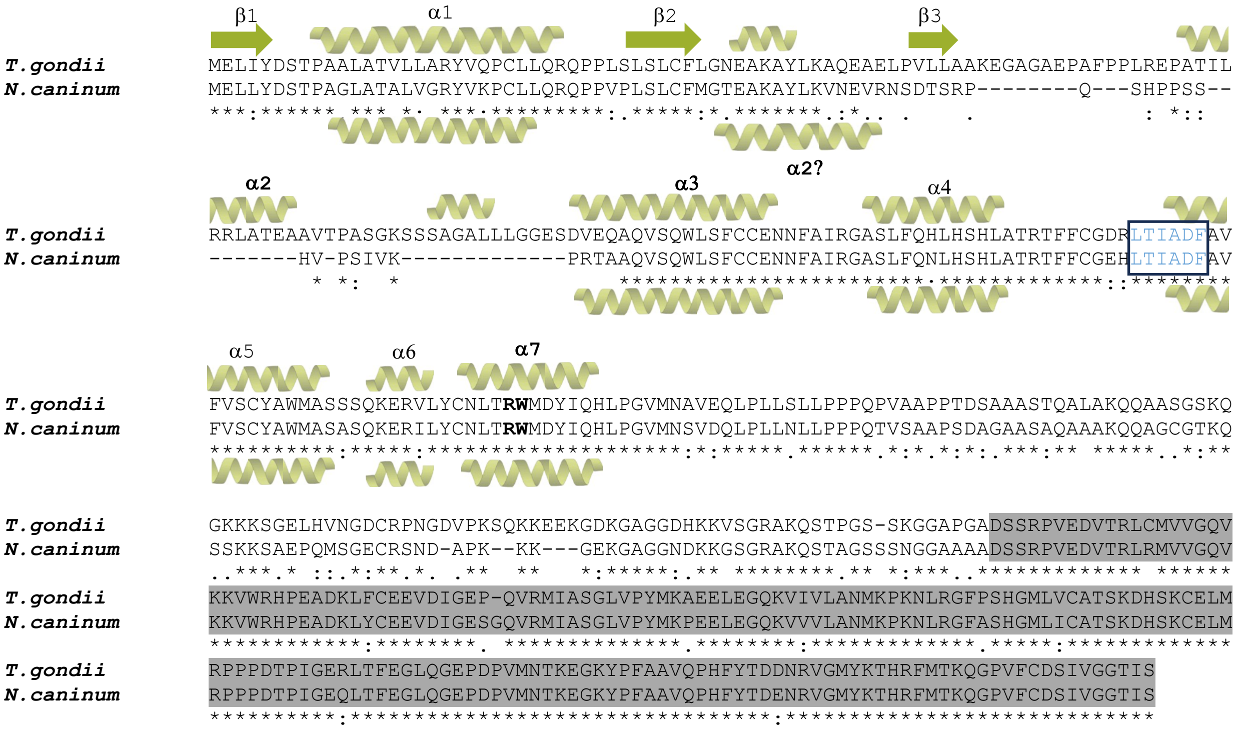

Phylogenetic tree based on full sequence alignments of *Apicomplexa* AIMPs

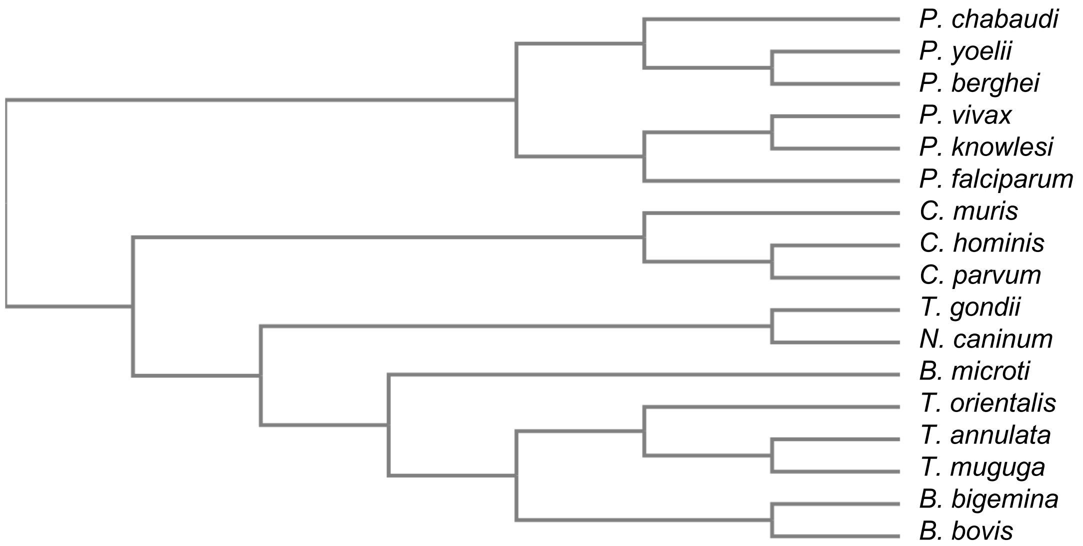

Figure S2

D GST-like ERSs

PLASMODIUM

```
                 β1          α1                    β2        β3
P.vivax         MVESDGINIYYGK-KYPFVCRTILNAHKSLRQKENPAG-GDHSAEE-KVKFVKDEN-VEELKVEFVS-------------
P.yoelii        MIKDSKINIYYGK-NYPFLCRTVFNIYQNNIKKKTANNLNDKKIEEICVNFINDKTIVEDIKIEFVK-------------
P.berghei       MIKDSKINIYYGK-NYPFLCRTVFNIYQNNIKKKTANNLNDKKIKEICVNFINDKTVVEDIKVEFVR-------------
P.knowlesi      MIGGDKIIIYYGK-KYPFVCRTILNVHKILSQKENVEGRSNHFPND-KVKFVKDEN-VEEIKVEFVN-------------
P.falciparum    MIENTKVNIFYGNNKYPFICKSILNVYKNKNKKILEKTETTVNVDE-NIKFIKDAN-VEELKIEFLTNAPNNNNNNNNNN
P.chabaudi      MIKDSKINIYYGK-NYPFLCRTVFNIYQSNLKKKTAN-LTDQNIEEVCVNFINDKKVVEDIKVEFIR-------------
                *:  .   : *:**: :***:*::::* ::    :*           .:   ::*::* .  **::*:**:

                                                      β4   α2
P.vivax         -----------------------------------KEEAQGEKCINTTDKIFAKSLDQILQTKLYHSF--RENNKKEDKQ
P.yoelii        ----------------------------NSVADESRKNVNTNNSVISSDKIFAINLDFLLKTNLYYFTSYRENINK-NII
P.berghei       ----------------------------NTVSDQTRKNTNTNNSVTSSDKIFAINLDFLLKTNLYYFTSYRENINR-NII
P.knowlesi      ----------------------------------KAEEENGDKCINTTDKIFAKTLDEILQTKLYHSF--CENNKKEDKQ
P.falciparum    NNNNNNNNNNNNNINKGNMNHCGKNQEEEKKNLSKSNNIEKVPYICSSDKIFAKILDHLLKTDLYNIS--EKIDPKNNKV
P.chabaudi      -----------------------------SSVSEEASKNPNTINVSSSDKIFAINLDFLLKTNLYYFTCYKEIINK-NII
                                                         :  ::*****   **  :*:*.**       :   : :

                     α3              α4                     α5               α6
P.vivax         TDVYVQAQYDEWVDYFRSKQLQKEVLIICDHLNRHLHLNTFICGDYLTLSDLYVYYEMHRYFNVANCYNVKYSKQFRNVN
P.yoelii        TNVFFQAQYNEWIDFLRNKDIEKNIIPICEHINKHLYLNTFLSFHYLTLSDIYIYYEMHKYFSGNITTNLKYPKQYKNIN
P.berghei       TNVFFQAQYNEWIDFLRNKDIEKNIIPICEHINKHLYLNTFLSFHYLTLSDIYIYYEMHKYFSGNITTNLKYPKQYKNIN
P.knowlesi      TDVYVQAQYDEWVDYFRNKHMQKEILIICEYVNKHLHLNTFICGEYLTLSDLYVYYEMYRYFNVANCYNVKYTKQFRNIN
P.falciparum    VNIYVQTQYEEWLDFFRSRNVNKDIVFICEHLNKHLHLNTFVVSEYLTLSDIFIYFQMYKYFHVSSTQYAKYSKMYKNIN
P.chabaudi      TNVFFQAQYNEWIDFLRNKDIEKDIMPICEHINKHLYLNTFLSSHYLTLSDIYIYYEMHKYFSGSITTNLKYPKQYKNIN
                .:::.*:**:**:*::*.:.::*::: **:::*:**:****:  .******::*::*::**        **.* ::*:*

                  α7        α8
P.vivax         RWFKLINCLVN---YSDAELSTMLKKEVETNQVDITK-------------DANTMQLLKQKIVSTSYV
P.yoelii        RWFKLIKSLLHDHITHDAELVQNLKIKEKIFNTTNYGNKKSGNNEDKSSTHPNKSNTKVGGTSLSSYE
P.berghei       RWFRLIKALLHDHVATDAELIQNLKVKEKIFNTAGSG-KRLGNNGNKYSAHSDHTNTKGGATSLSSYE
P.knowlesi      RWFKLINCLVN---YPDEELSTMVKKELEKNQVNITK-------------DANSKQLLKQKTVCTSYG
P.falciparum    RWHKLIDSLIY---FEDAEWKKNLKCLICDNKMNFDDRKDNLKNNNKSMNNTDDVKC-KKKTVTTSYS
P.chabaudi      RWFKLIKSLIHDHISTDAELIQHLKIKERMVSTGGAG-RRPGNNADKSSGD-------HGKGSAASYE
                **.:**..*:       * *    :*      .                   .           :**
```

THEILERIA

```
                     β1           α1           β2        β3  β4    α2
T.annulata      MYNKGSEQLVVNYSVQNPPYGAVILSSISKYLQKSDSSKVVFNLDNKLQNNEFSLNGDKFNEVDLLKNLCSVLPYCDKLL
T.orientalis    MKASNPCSLVVNYSVQNPPYGAVILSCISKYLQKSESSPVEFNLDNKLQNNEFSLNGNNLNEVELAKKLTTLLPYCDKLL
T.parva         MYSKGSEQLVVNYSVQNPPYGAVILSSISKYLQKSDSSKVVFNLDNKLQNNEFSLNGDKFNEVELLKNLCSVLPHCDKLL
                *   ... .*****************.********:** * ****************:::***:* *:* ::**:*****

                     α3                    α4                  α5        α6
T.annulata      DVVNNSELEYWFKVLLSEKSKLAENKGNFTENDSYLENMNKHLAQRTYLIGHRLSLSDILHFSLLRRTCNVNSLKNKYPH
T.orientalis    DVPEYSELDYWLKVLLLEREKHLETKKAFFENVEYFEQMNNHLAHRTYLIGYRLSLSDVVHFSFLRSNCNVNSLKNKYPH
T.parva         DVVNHSELEYWFKVLLSERAKLTENKGTLAENDSYLENMNNHLAQRTYLIGHRLSLSDILHFSLLRRTCNVNSLKNKYPH
                ** : ***:**:**** *: *   *.*  : ** .*:*:**:***:******:******::***:**  .************

                  α7
T.annulata      LARWYNFISNVP-GTSGCLNGYETPSSATYKKPFTKQEQQDNSYK
T.orientalis    LVRWYNFISNVPVGTYECLKKYDAPARVQYKKPFTKQEQQDNSYK
T.parva         LVRWYNFISNVP-GTSGCLKGYDTPSSATYKKPFTKQEQQDNSYK
                *.********** **  **: *::*: . ****************
```

BABESIA

```
                     β1           α1           β2        β3  β4      α2
B.microti       -----MYEFNVLYHPTCTILG---IDAFAN-LTRKEGIKVTLICDKGANVNSYTIKSRDSILTESFSCELSALDYLSGWS
B.bigemina      MAETTRDAVTVVHAPRETPYGAIVLAAIAKRFSKANESPIAFEAGKAIEPGTYLLEK-AKLQEPQLLRELCLRIPSCKRM
B.bovis         MDNGSKCAVTVQHAPKDVPFAAIVIDAIAKQLSRSDDDVVKLEQNKAITPGTYVLDK-VSMQISQLVRELCLRVSTCKRM
                        ..* : *  .  .    : *:*: ::: :    : :  .*.    .:* :..  .:   .:   **.      .

                      α3                 α4                    α5          α6
B.microti       KNFQPQENIEIWAEILTNCSKDETLM--------AALDKYLSLKTFLSGHSMSCIDIAMFSIL-------KPDPPYPNVT
B.bigemina      LYIDSSASLDQWINYVTLHSATPKTPVADLWQYYNKMNTHLATRTFLIGYRMTVVDVLQFAALSNAKAVCKELAKVPHVQ
B.bovis         LYIDSTASLDKWLSYISEGAGMHKT-EEQLWQHYTKINQHLTMRTFLVGYRMTAVDVLQCSLLANSQGIRKRLTELKHLE
                  ::.  .:: * . ::  :   .                 :: :*: :*** *: *: :*:    : *         *  .  ::

                 α7
B.microti       RWHRFIAHLIGPTISTN-----D-------KFGNQGNVRKAQVDNSYR
B.bigemina      RWYNYIIAIPGVQQAVS-----DIQRKNVPQVEKKPFTKEQQIDNSYK
B.bovis         RWFNYIMAIPGVNSNPGTATTNRAENKVFTKNDKKPFTKAEQFENSYK
                **..:*  : *     .                 :  ::  .:  *.:***:
```

Figure S2

TOXOPLASMA / NEOSPORA

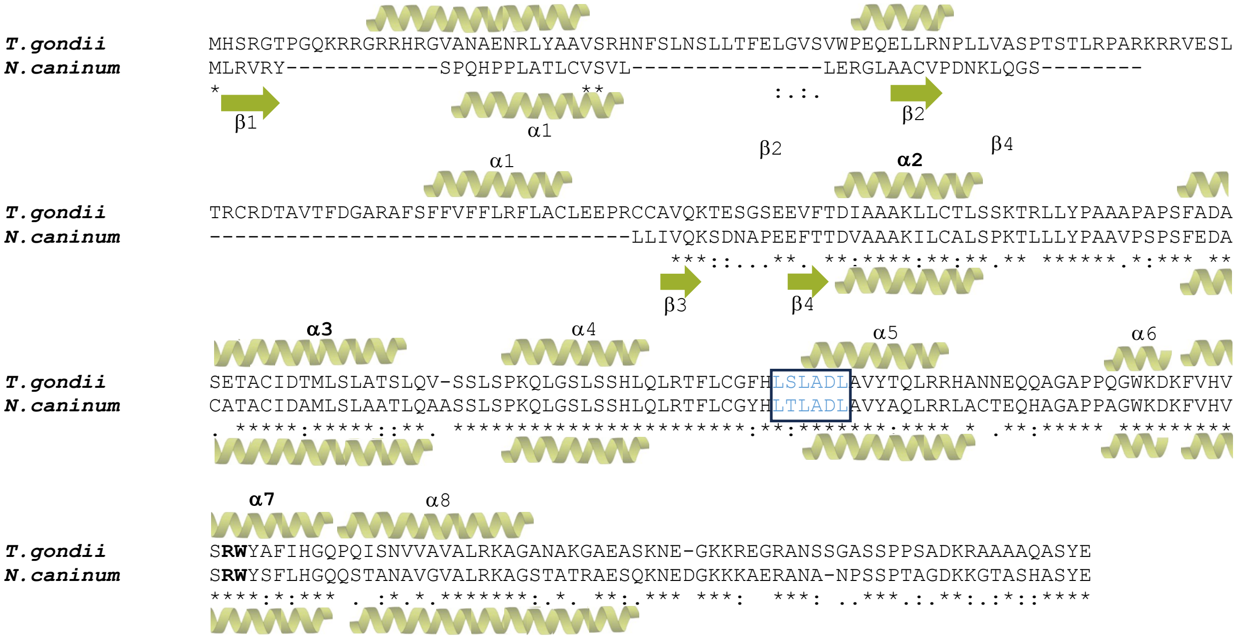

Figure S2

E GST-like MRSs

PLASMODIUM

```
                   β1          α1          β2                          β3     β4       α2
P.vivax      ------MILYPHKYFPN-TVKCMLAAHIYSFQVELCDDFFYTRSFDIEKSLVVNKKPLLVYEDNYVSSTKAICFLFHRLR
P.yoelii     ------MILYPNKYLLSGTLKCMLAAHIYKLPVELSDDFFFTRTFEVDKSLIVNKKPLLIYENKFISSTNAICFLFHRLK
P.berghei    ------MILYPNKYLLSGTLKCMLAAHIYKLPLELSDDFFFTRTFEIDKSLIVNKKPLLIYENKFVSSTNAICFLFHRLK
P.knowlesi   ------MILYPHKYLPN-TVKCMLAAHIYSFPVELSDDFFYTRSFDIEKSLVVNKKPLLVYENNYVSSTKAICFLFHRLR
P.berghei    MMKPLNMILYPHKFFLN-TVKCMLAAHIYNFNVELSEDFLYTRTFDIEKSLIINKKPLLIYENNYVSSTKAICFLFHRLR
P.chabaudi   ------MILYPNKYLLSGTLKCMLAAHIYKLPVELSDDFYFTRTFEIEKSLVVNKRPLLIYENKFVSSTNAICFLLHRLK
                   *****:*:: . *:**********.: :**.:** :**:*:::***::**:***:**::::***:*****:***:

                                   α3                  α4
P.vivax      NVE-SKKCVDEKLRLYMSWVEWSDLLEKHIEVLNKKKIIDSLDELESYLKENP--NKNFICSL----------GEATQGD
P.yoelii     NKGINTKGLDDKLRLYLTWIEWSDILEKNIERLNKKKIIESLDLLESYLSENK--NKKFICDEVDQIKREDECSETDQNQ
P.berghei    NKGINTKGLDDKLRLYLTWIEWSDILEKNIEHLNKKKIIESLDLLESYLSENN--NKKFICDEVDQIKREDGCNGANQNP
P.knowlesi   NAE-SKKCVDKKLQMYMSWVEWSDMLEKHIEVLNKKKIIDSLDELESYLKENQ--NKNFICSC----------EQDSQGD
P.berghei    NK--SKKPFDENLSYYLGWLEWSDLLEKHIEVLNKKNIIESLDELELYLKENKNKNKNFI--------------NNDDEK
P.chabaudi   NKGINAKSFDDKLRLYLAWIEWSDILEKNIERLNKKKIIESLDVLESYLSENN--NKEFICDDAEQIKREDGCDETDQSQ
             *   . * .*.:*  *: *:****:***:** ****:**:*** ** **.**   **:**                 :

                       α5                      α7                 α8
P.vivax      SND--KLTLADIFVYTSLTHCNIEVCRESWAHISNYIEKINQLEDVQKILKDVDRIYKYKNIYNLFISKIHEKNINTYLK
P.yoelii     KNDICGITLADIYVYTSLKYSNIVICNESWSHINNYIERIHNLEDVQKVVETVEKVHKLKNIYSLFISKVCEKNINDHVK
P.berghei    KNGICGITLADIYVYTSLKYSNIVICNESWSHINSYVERINNLEDVQKVVEAVEKVHKLKNIYSLFISKTCEKNINDHVK
P.knowlesi   GND--KITLADIFVYISLTHCNIEICGESWIHINEYIQKINQLEYVQKILKDVEHIYKYKNIYNLFISKIHEKNINSYLK
P.berghei    DEN--KLSLADIFVYTSLKHSCIEICKESWVHINEYIEKINNLQEIQNVIKNVEEIYKYKNIYSVFISKIHDKNVNEYLK
P.chabaudi   ENGTCGISLADIYVYTSLKYSNIVICNESWSHISSYVERINNLEDVQKVIETVEKVHKLKNIYSLFISKICEKNINDHVK
              :.   ::****:** **.:. * :* *** **..*:::*::*: :*:::: *:.::* ****.:****  :**:* ::*

P.vivax      EEKFYI
P.yoelii     NDNFYI
P.berghei    NDNFYI
P.knowlesi   KEKFYI
P.berghei    NETFYI
P.chabaudi   NDNFYV
             ::.**:
```

THEILERIA

```
                  β1       α1          β2
T.annulata     MEHHLLILNDSNESHLCRFLLKTLEPSFPLVVLYGPKVPDKLSDLYIESSELEHLKDSTTDSNNYKYSVLYKELNKSNSL
T.orientalis   MEYHLLILNKSNESLLCRNFIKIWASSAPVVSLFSSKIPEDLSDIHIDSDDHDTFKDSTSEANSYKYQQLYNELNRLCTL
T.parva        MAHHLLILNDSNESQLCRFLLKTWEPSFPLVVLYGPKIPDKLSDLYIESSELDHLKDSATDSNNYKYTVLYKELNKLNSL
               * :******.**** *** ::*   .* *:* *:..*:*:.***::*:*.: : :***::::*.***  **:***:  :*

                                                     α2                           α3
T.annulata     THQEFEELYNNLVKYLKTVLFRPVLLLFKKSNNFLPNVLFNFKPILLYLSPLYNLHKVDGQKKQYREDKNLLYWYLEFDY
T.orientalis   SQQDFSIIHTNLFKYLKRALFRPLLLLFKKDNEFVPNIVFNLKPILLYYYPS--LRKVSDTQ---KEDKELLYWYTEFHS
T.parva        TQQEFEELYNNLLKHLKTVLFRPLLLLFKKTNNFLPNVLFNFKPILLHFYPLYNFPKVDGSNNTYREDKNLLYWYLEFDY
               ::*:*. ::.**.*:** .****:****** *:*:**::**:*****:  *   : **.. :   :***:***** **.

                          α4                    α5                        α7
T.annulata     NLNNIGTNNRLLDLLDELENTIEEYNLDNKFVQSILSLLNMYVDRILFRNISLTKYTKVLKYSKQYSTLLNT--------
T.orientalis   NFNYVGTNRKLVELLDDLEENLSRYNLESPLTQLLLSTLNMYIDGLLFRNIALSKYTNILRQATHHKHLLKNSLLNKFNY
T.parva        NLNSIGTNNRLLELLDELENTIEEYNLDNKFVQSLLSLLNMYFDRLLFRNISLTKYTKVLKYSKLYYSLLNT--------
               *:* :***.:*::***:**:.:..***:. :.* :** ****.* :*****:*:***::*: :. :  **:.

T.annulata     ----HLSSTGTYTHA--------KGS-------TTE-AQETS-------------------SKFTEKGEVGHE-----EL
T.orientalis   QGTATVSATGILSPSIPTSLSGFKGSDKANGASKGK-SESAKSPLKGSGKGAESSSVYNMGSGKEGKEDAEKEWEPGLER
T.parva        ----HLSSTSTYTPP--------KGS-------STPSTQDTG-------------------SKMSENPEVGPE-----EL
                    :*:*.  : .         ***       .   ::.:                    *    : :.  *     *

T.annulata     DKSSESADKLNLVDYK
T.orientalis   DDDLEEDEKLSLNDYK
T.parva        DKSSGAPDKLSLVDYR
               *..     :**.* **:
```

Figure S2

BABESIA

```
                      β1        α1          β2
B.bigemina   MQ--LLVLDSSIDSLVTWSAAECVCDSVPTVVLASISTESDLRSNKCRNEVHALKCLQELPLLCSKLESQDAHSVKLFTE
B.bovis      MQ--ILILDQSLESLAAWLTAEFLRNSSPLALLSSVTRDTNWNPSASFEEHNGYKCVNTLSLSCENFEQNDGDFHGFLES
B.microti    MQYTLLLDPSCYHSFACYLASAQFNFPAKIEIAADVNIE-----NRLVSEEYNLSKFKDKKLQIATLDT-----------
             **   :*:   .. .*:. : ::   .   .     : :.:. :        .    .*     . .:    *    .::

                                                        β3        β4      α2
B.bigemina   HIGKHTSQPSEDVLKH---L-LKTLNEVPVRPLLFAVDSSVADVLPIFGCNAVLKYYVEGQGVCGKSSCDLYTPTYEQSA
B.bovis      IFGLNPEACDTSTVKSTKAIIVQAQKDILVRPLLLIVNRNNLEVSPIFGSDSILRYYLENQTSTINGNNILLNSEQVKNH
B.microti    ------------------------------KPVLIVNIGN--DLIKIKGIRAILLYIYRY------------TIKDIDNF
                                           :*:*:     .   ::   *  *   ::* *    .             .    ..

                α3                    α4                         α5                   α7
B.bigemina   FDWFSWTSQLRLLCKKNASVDLRALLSQFDAYLHGQQECDIKVGIGSVVITLCLLWYETKLEFLGCNLDMFSHIRQYISA
B.bovis      FDWKSWSSTLRTVCRKNNSDLARGLLSSLNAFLTSNIDLICYDIIGSAIITLSILWFNGKIEALGLRLDMFPSIPPFIHN
B.microti    YNIYHYFD----------------LLERLEALLTGVNKLHIDNILQK---------LDDHLSTSDYLLDKITPVDIYLYS
             ::   : .                **. ::* *  .   .      : .         : ::.   .   ** :. :  ::

                             α8
B.bigemina   VLTATANVERYRCLHTFYRLHGTCAAPERSLASSRELE--------SLCRDFIDR-KFYV
B.bovis      VLTKTSSLNKARCLHLFYRSMETSRSHVNGNGALKSLS--------DVSRDLIGK-KYYI
B.microti    MLSAMHNFH-WKNLSKFHSNLHMFANHLSKFKLTQQFYDYSTQPYDNLSVDIKKCKNYYI
             :*:    ...  : *   *:                 :.:           .:. *:    ::*:
```

TOXOPLASMA / NEOSPORA

```
                                   β1              α1        β2
T.gondii     MSTMTGSSPKSAASSTPLRLVAGSAVPRGDVAACIAVGHALNIPVVLVSKDADKAEQAEPSSSAPAPPPQAPVASSASEE
N.caninum    ---MTDSA-PSGASSSPLRLLAGCTVPRAEVAACLAVANALKVPVVLSSKDAAES-KARPSQASLAPTPHAPASDPTVDE
                **.*:   *.***:****:**.:***.:****:**.:**::**** ****  ::  :*.**.:: **.*:**.:..: :*

                   β3     β4             α2                                 α3
T.gondii     DKKDLCVLLTREGSVVSAYAICLYLLSLARKRLAETKTAAP--EKRLESREKLSEAYDGDAWQAEDALAWAAFKLRGALR
N.caninum    DKKDACVLVTPDGSVVSAYAVCLYFLSVARKRRQAEKAAPQQEKRQEGEAAPARDAERDAWQAEDALAWAAFKLRGALR
             **** ***:*  :********:***:**:****    :::.***   *** *..     :.    : *********************

                      α4                                   α5                 α7
T.gondii     GVRKDLLLQQLNLLEERIAG---SSDCAFPALSALLSTSAPSPFVAPAMFVFCMLRYAHPTSAFSDWPALSAFLGSLGAS
N.caninum    GVRKDRLLQQLNSLEERLSGDPQTSPAAFPSVSSLLAA-SPSLFVAPAMFMFCLLRYAHPTSSFADWPSLAKFLQALGSS
             ***** ****** ****::*    :* .***::*:**::  :** *******:**:********:*:***:*: ** :**:*

T.gondii     PAAAATLKAVPLPARSRGFPEMSQALLASAGACAVAQRQREKFYI
N.caninum    PAVAATLHAVPLPSRACGARALTRALLSSSA-SPQTEKTRERFYI
             **.****:*****:*: *    :::***:*:. .. ::: **:***
```

Figure S2

F GST-like QRSs

PLASMODIUM

```
                      β1               α1                β2       β3       β4      α2
P.vivax         MENVEKIYIRNKAELSDNFFYFYNWVKLNFNTCLIISENSSLRSSVLLELLNGKKLVLSEINVCEALFRLACGGI-----
P.yoelii        MEHIEKIYIKNKSDLGNEFFYFYNWVKEKFNISLVISESNSLRHNIVLELSDGNNLSLNELNSCEAIFHLISNDK-----
P.berghei       MEHVEKIYIKNTSDLGNEFFYFYNWIKEKFNISLIINESNSLRHNIVLELSEGNNISLNELNSCEVIFHIIRNSH-----
P.knowlesi      MENVEKIYIRNKADLSDNFFYFYNWVKLNFNTCLIISENSSLRSSILLELLNGKKLVLSEINACEALFRLTCGGV-----
P.falciparum    ---MEKIYSKNKLDYLSIIFNFYSWYKRHFKGTLLINENSNIKNTTLIELLGNKKLLLNEINACEAILKLNEHHENYDSS
P.chabaudi      MEPIEKIYIKNKSDLGNEFFYFYNWIKEKFNTSLIISESSSLRHNIVLELPEGYKLPLNELNSCEVIFRIICNGY-----
                   :**** :*. :  . :* **.* * :*:  *:*.*...:: . ::**  . :: *.*:* **.::::

P.vivax         -----------SGE---------------------------------------SSGGVSG-----------EISDVSCHH
P.yoelii        -----------NSKNS-------KN-N---------------------QNNKHIWNNEFE-----------SEQDVEENP
P.berghei       -----------NKK---------------------------------------DNWGNEFE-----------NEQDVEENP
P.knowlesi      -----------SNAVG-------GTVS--------------------CTVNCLSNGPVSG-----------AQRDDFANR
P.falciparum    SGTCNVNNINQPNNNNNNKENINDNNNNNKENINDNNNNNKENNINPCSTNSFNINSFSTNNYNNFNNPCCSFSCNIYDY
P.chabaudi      -----------NKN---------------------------------------NIWGNEFE-----------NEKDKEENP
                                                       .                       .

                       α3                    α4                  α5
P.vivax         RIDALYAEELKFFLKGRLASDKPVDEQFLERVEAEMALQ--KGFCKER---LSFLDFLLFSIFKKAKVDQVEG--NKYPH
P.yoelii        -FRISQIEELKNFLKNILLQKKKLENNFYERIEKEMMNKNNNIFSKDK---LRYIDFLLFCILKKNKIEKIN---NKYEH
P.berghei       -FKIVQIEELKQFLKNILLQKKKLENDFFEKIENEMIKKNNNIFAKDK---LRYVDFLLFCILKKNKIEKIN---PKYEN
P.knowlesi      CRDTLRGEELKFYLKEKLASEKPVDEQFLQRVEAEMALQ--KVFCKDK---LSFLDFLLFSIFKKGKVEKLKDPLGRYPH
P.falciparum    -YDILKIEELKDFLKDFILRNKKINKEFFDTIEEDMTMWKKIFFFGQKKRTPEFLDFLLFYILKKENIHFFEE-ETRYIN
P.chabaudi      -FKIVQIEELKHFLKDTLLQKKKLDDNFFEKIENEMICKN-TIFSKDK---LKYTDFLLFYILKQNKIEKIN---PKYEN
                       **** :**  :  .* ::.:* : :* :*       *  ::     : ***** *:*: ::. .:    :* :

                α7
P.vivax         LGAWLSKNN---FP--IKEEDMNSFAS
P.yoelii        LNNWYLQNN---IS--VKEESMNSFTN
P.berghei       LNNWFLKNN---IS--IKEESMSSFTN
P.knowlesi      LSAWFSKNN---FT--IKEEDMNSFAS
P.falciparum    LHNWFLKNKIYDISNDKKEDNTPSACN
P.chabaudi      LNNWFLKNN---IS--IKEESMNSFTN
                *  *  :*:   :.   **:.  *  .
```

TOXOPLASMA / NEOSPORA

```
                          β1                α1                β2            β3
T.gondii        MALSPGAVLRITRLASLPPLSTVTDVVLAYIEQNLKSAGDFTFSVQPDTSSTPAAPFRIAVETKKGEQPSPIAAGEALSD
N.caninum       MALSPGAVIRVTRLSSLPPLSTVTDVVLSYLEQHLKSAGGFTFSVYPDTSSSPATPFRIAVETKKGEAPRPIAGGEALSD
                ********:*:***:*************:*:**:*****.***** *****:**:************ * ***.******

                    α2                       α3                          α4
T.gondii        YAAARCIARLAASTGDPFASLLKASGAPGGVEAAVDVCEQWAAFYGVSGKAPEASDNPGLSACIDFFARRFSTSSSFLFG
N.caninum       YAAARCVARLAASTGGVFACLLNENGATESADGAVDACEQWAAFCGVPGKTPEAAEHPGLTACIEFFTRRFSTSSAFLCN
                ******:********. **.**: .**. ..:.***.******* **.**:***:::***:***:**:*******:** .

                       α5           α7
T.gondii        TPQPSLADMTLFALLKAHPAAQTALSSALRTSETAAKHFSSWSACVAKAIPGAGALSGAGAKGTDRAKGKSGEMVG-QGA
N.caninum       TPNPSLADMALFALLKTQPAAHTALLSALRTSGEAAKHFSAWSSSIAKALPGATTLSGAGVPD-GSAQGNRGEISGATGA
                **:******:******::***:*** ******  ******:**:.:***:*** :*****. . . *:*: **: *  **

T.gondii        GVASEAVDGPDATTS
N.caninum       GASPESTDGPDATAS
                *.:.*:.******:*
```

G N-terminal extensions of YRSs

```
P.yoelii        ----------------------------------------------------------------------METINIKKED-NE-----------AQVKISQEIH-DENVEKRYNEIMSITSEC
P.berghei       ----------------------------------------------------------------------METINIKKED-NE-----------AEVKISQEIH-DENVEKRYNEIMSIASEC
P.knowlesi      ----------------------------------------------------------------------MESDGVKREE-LQGATGEAAGLVEAQETAPQENPPQEDVSRRMTEILSIASEC
P.falciparum    ----------------------------------------------------------------------METTDTKREE-QE-----------IEEKKAQEESKIEDVDKILNDILSISSEC
P.chabaudi      ----------------------------------------------------------------------METINIKKED-NE-----------AQVKIPQEIH-DEEVEKRYNDIMSITSEC
P.vivax         ----------------------------------------------------------------------MEGEGVKREE-LEGATGEAAPQAASQEAASQEATPQEDVSAKLADILSVASEC
T.annulata      -----------------MSERFISPFHGGIAVNYS------GNVVN-----------VKNPNLEFHHDSCSIKCGLREEL---------DRYFRGS----RSQMDLTAEEKVKICLSLSEECI
T.orientalis    MLISIKNCNLSI-RLKNMSERFLSPFHGGIAVNYS------GNVAN-----------IKNPKLEFYPGSCSVKHGLRKEI---------DNFFHKS----SFHGNQSLEERVNTCLSMAEECI
T.parva         MSIYVKKSSLCIFRFKKMSERFISPFHGGIAVNYS------GNVVN-----------VKNPNLEFYNDTCSIKYGLREEL---------DRHFRGS----RPQMDVSVEEKLKICLSLSEECI
B.microti       ML---------------RFRMALSQFTGGL-YKGEIHNPLYGSKIK-----------LKQTICAFTPSQFYLEGGIRKNI---------DKYFEGK----L-STKLSVKERLDLCLQIGSECI
B.bigemina      -----------------MSRVFASPLHGGLEFDGE------EECYK------------TSTGFEFHPGSCAAKNSIRERL---------NAHFAPS----LAEPRIPVAEALERACKLSVECI
B.bovis         -----------------MMRSFVSPLHGGLDLEGT------ERSHR------------TRTDFEFYPKSCAIKNGLREEL---------ENHFRGM----QIESQLSLKDAIDICNQMSVECI
```

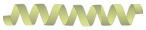

```
T.gondii        ---------------------MASPFTGGLSVW-DDSLLT-SHVASVSTAASTS-SPPA---NEASNATPATKRGEKEKKTSGGVTTLYDSIVNGAQWPQPRPSSLPLDERVQLCLSIAQECI
N.caninum       ---------------------MASPFTGGLSVW-DERLVS-SQLAALSTTASPSPAPPAPANQEANSAAVSEKQGGKDKKSSGFV-TLYDAVVNGDKWPAPRPSSLSLEEKYQLCLSVAQECI
                                       * : **:                                          : . :..          :  .             .  :  . . .:. ***
```

Figure S2. Sequence alignment of GST-like domains present in *Apicomplexa* EF1 and multi-synthetase complexes. Protein alignments were performed with M-Coffee [22]. The corresponding EupathDB annotations are given in Table S1. The complete sequences of AIMPs are shown with their EMAPII-like domain highlighted in grey. For EF1 subunits and aaRSs, alignments were performed on the full-length protein sequences, but only the GST-like domains involved in protein-protein interactions are shown. The RW motif in helix α7 and involved in interface 2 is highlighted in bold. The N-capping box (φ-S/T-X-X-D-φ) is shown in blue in the central helix α5, when present in the sequence. The GST-like domain of EF1β is reduced to only 3 α-helices including α5 and α7 which are sufficient to form an interface 2 with EF1γ. The strains used to run ColabFold modeling are indicated in bold and the corresponding secondary structures are displayed above the sequences or below for *N. caninum* proteins.

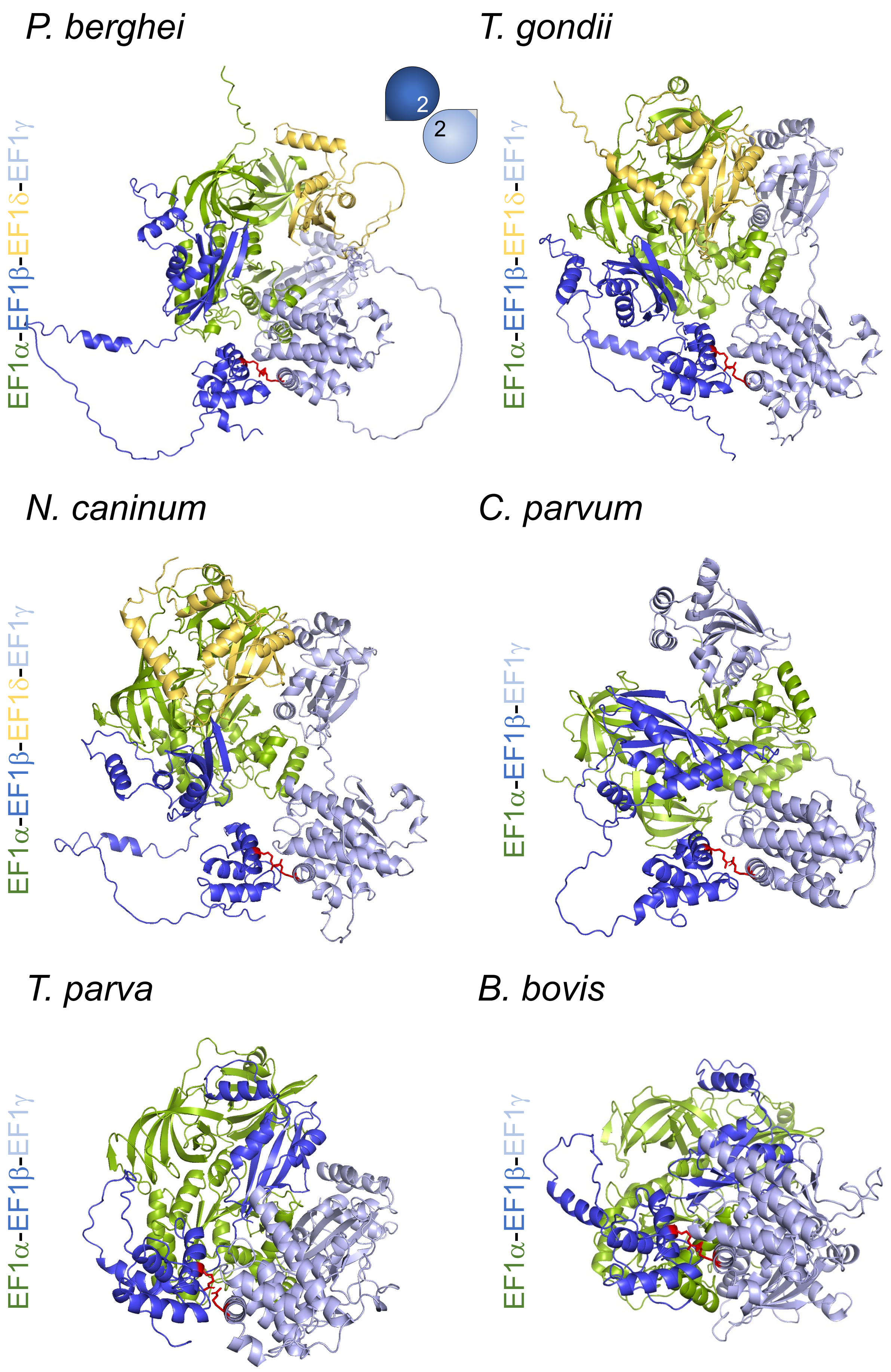

P. berghei
2
2
EF1α-EF1β-EF1δ-EF1γ
T. gondii
EF1α-EF1β-EF1δ-EF1γ
N. caninum
EF1α-EF1β-EF1δ-EF1γ
C. parvum
EF1α-EF1β-EF1γ
T. parva
EF1α-EF1β-EF1γ
B. bovis
EF1α-EF1β-EF1γ

Figure S3

Figure S3. Similarities between EF1 complexes in the *Apicomplexa* phylum. Molecules are colored in blue for EF1β, light blue for EF1γ, green for EF1α, and yellow for EF1δ. All models are shown in "cartoon". GST-like domains are schematized with drop shapes, with the same color code. *Plasmodia*, *Toxoplasma* and *Neospora* complexes are tetramers while EF1 is a trimer (EF1β, EF1γ, and EF1α) in *Babesia*, *Theileria* and *Cryptosporidum*, due to the impossibility to identify subunit EF1δ in the corresponding genomes. All EF1β-EF1γ heterodimers involve interface 2 shown in red; the parameters for each AlphaFold model are shown in Figure S4.

EF1

predicted LDDT per position

predicted alignment error

Plasmodium

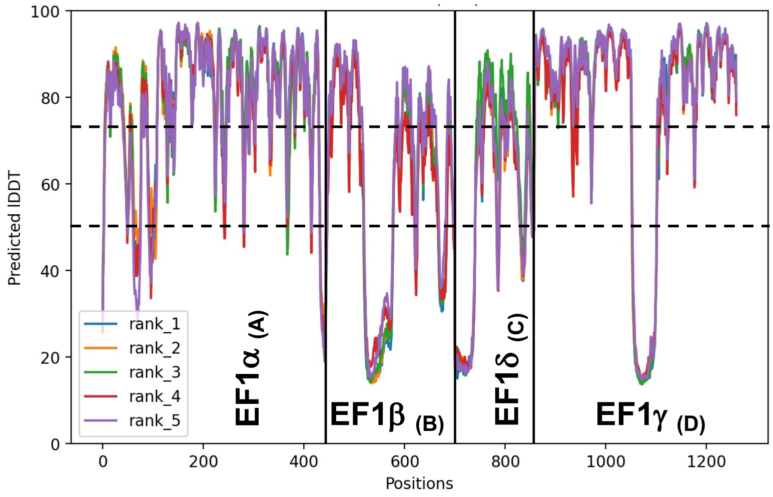

pTM
Rank_1: 0.44
Rank_2: 0.43
Rank_3: 0.44
Rank_4: 0.48
Rank_5: 0.48

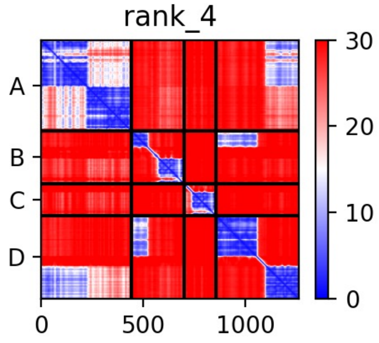

Toxoplasma

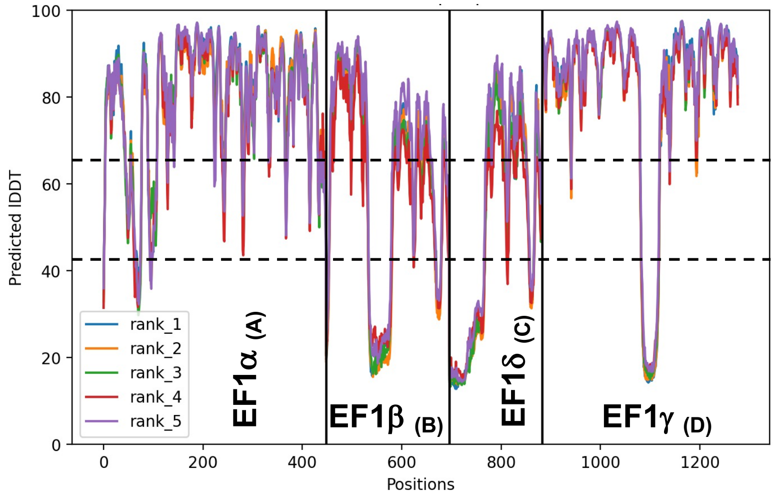

pTM
Rank_1: 0.45
Rank_2: 0.41
Rank_3: 0.5
Rank_4: 0.47
Rank_5: 0.48

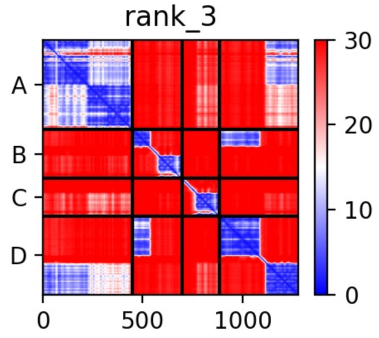

Neospora

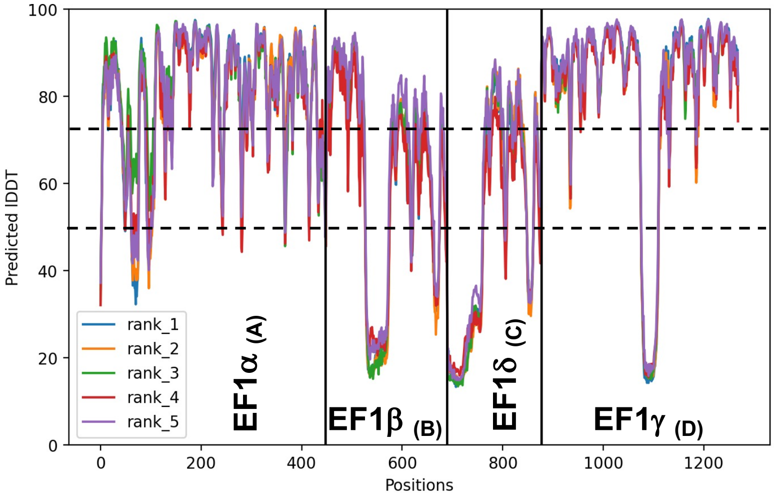

pTM
Rank_1: 0.45
Rank_2: 0.42
Rank_3: 0.50
Rank_4: 0.45
Rank_5: 0.49

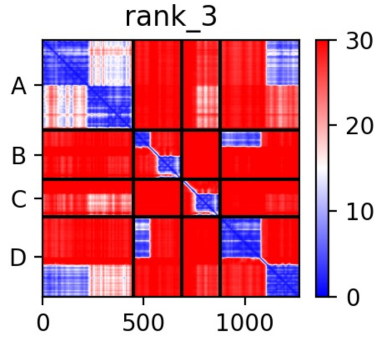

Figure S4

EF1

predicted LDDT per position | predicted alignment error

Cryptosporidium

pTM
Rank_1: 0.48
Rank_2: 0.49
Rank_3: 0.56
Rank_4: 0.58
Rank_5: 0.57

Theileria

pTM
Rank_1: 0.51
Rank_2: 0.47
Rank_3: 0.59
Rank_4: 0.59
Rank_5: 0.57

Babesia

pTM
Rank_1: 0.51
Rank_2: 0.52
Rank_3: 0.63
Rank_4: 0.74
Rank_5: 0.6

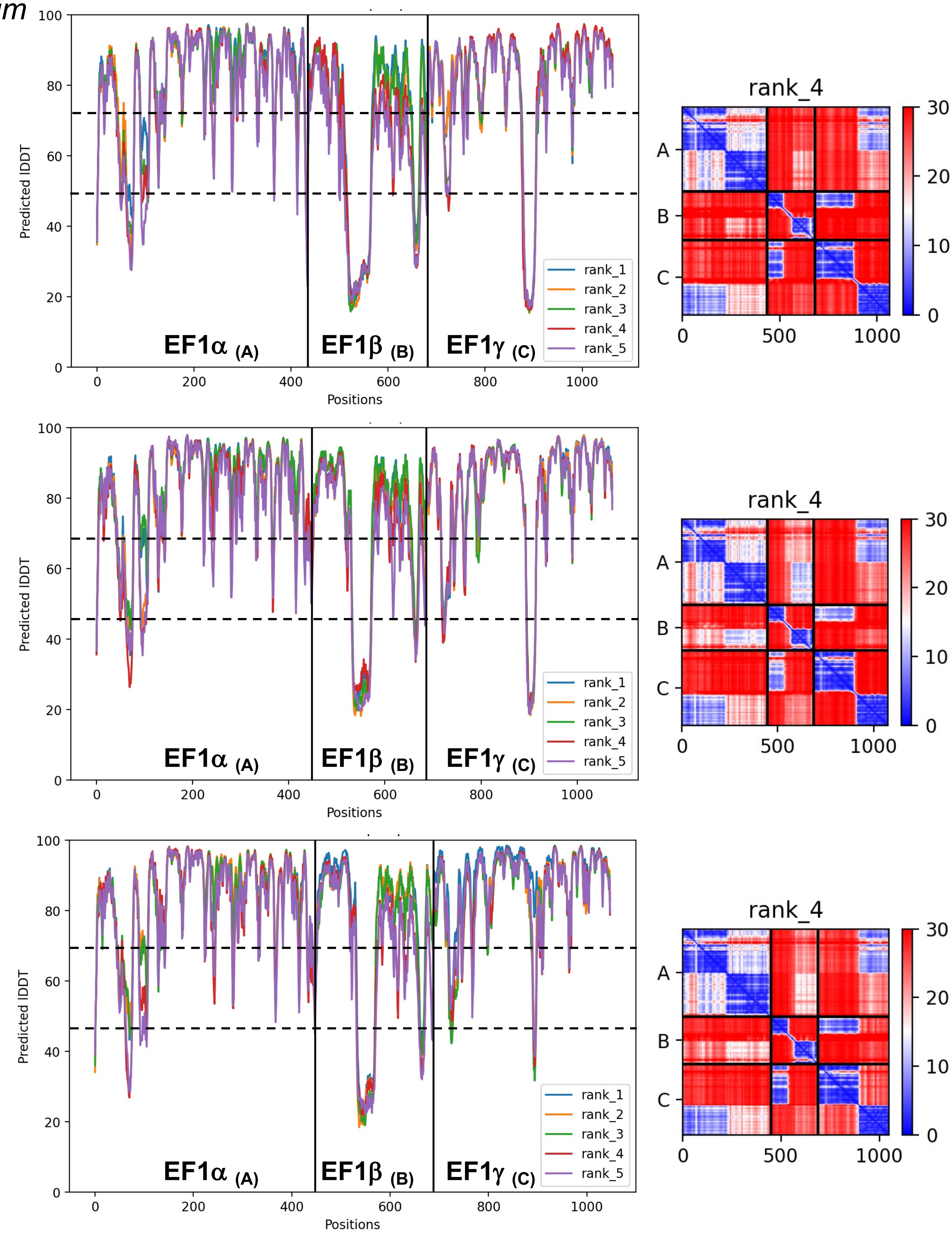

Figure S4

Figure S4. Confidence metrics of the ColabFold predictions for EF1 GST-like backbones. Models were ranked by pTM (predicted Template Modeling) scores, which measures the overall topological accuracy of the predictions. Predictions with pTM scores higher than 0.5 and showing the known GST-like interfaces 1, 1' and 2 were considered relevant and are highlighted in bold. The central plots show the pLDDT (predicted Local Distance Difference Test) scores per position. Regions predicted with high confidence are identified by pLDDT > 70, while disordered regions are identified by pLDDT < 50 and are presented as long filaments (not shown in graphical representations). The PAE (predicted aligned error) plot on the left indicates that the relative positions of domains are well predicted (blue).

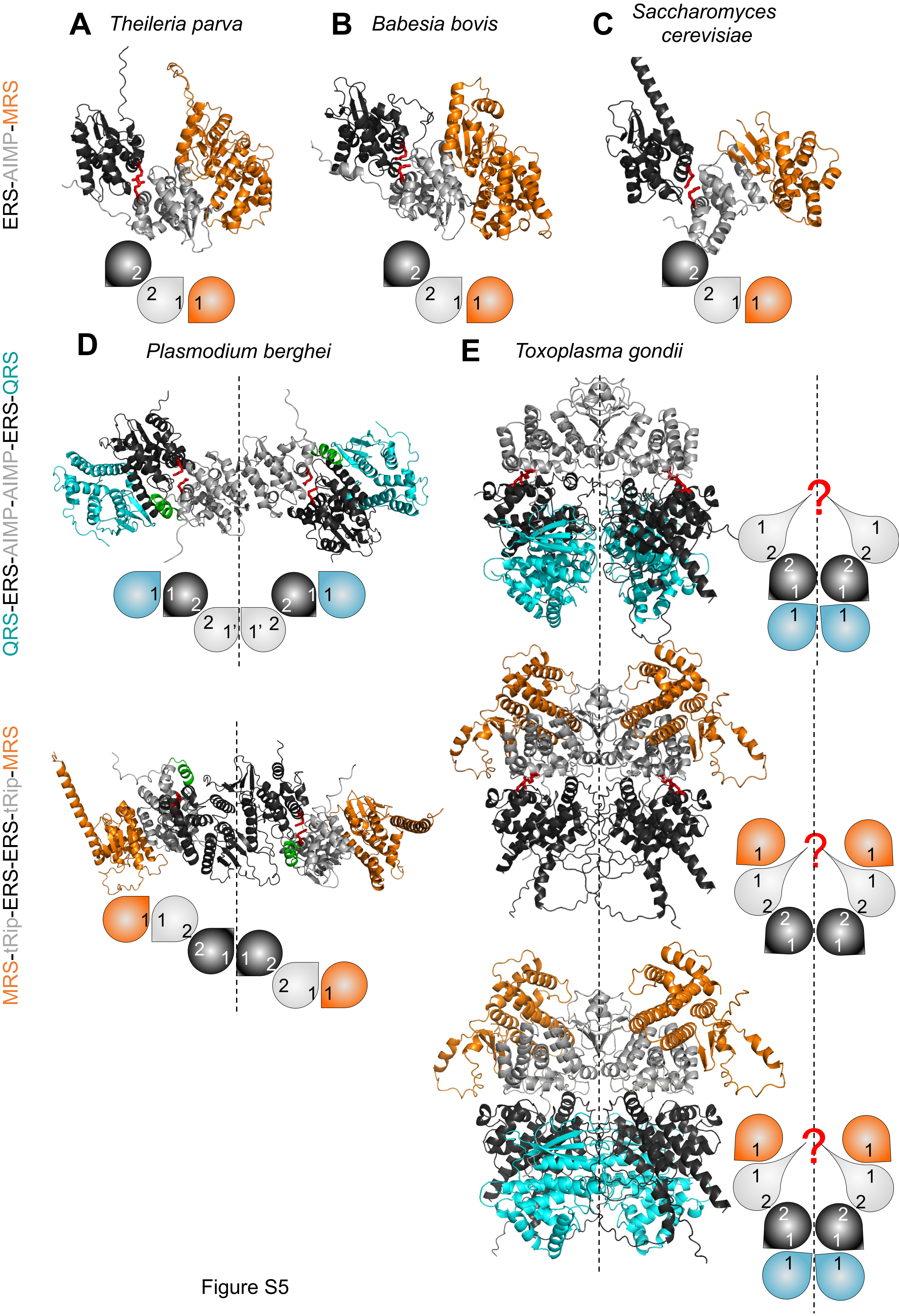

A
Theileria parva
B
Babesia bovis
C
Saccharomyces cerevisiae
ERS-AIMP-MRS
D
Plasmodium berghei
E
Toxoplasma gondii
QRS-ERS-AIMP-AIMP-ERS-QRS
MRS-tRip-ERS-ERS-tRip-MRS
?

Figure S5

Figure S5. Diversity of multi-synthetase complexes in the *Apicomplexa* phylum. All complexes are shown in "cartoon" with the same color codes and schematized below with drop shapes colored grey for AIMP, black for ERS, blue for QRS, and orange for MRS, all displaying interfaces 1/1' and 2. In *Theileria* (**A**) and *Babesia* (**B**), MSCs are organized into AIMP-ERS-MRS trimers similar to the Arc1p-ERS-MRS trimer determined for *S. cerevisiae* MSC (**C**) [28,49]. (**D**) The M- and Q- complexes of *Plasmodium* were determined experimentally [17,18]. They both involve equivalent GST-like domains but organize differently. In the Q-complex, symmetry is imposed by the tRip homodimer (interface 1',[13]) that binds ERS via interface 2, which in turn binds QRS (interface 1). Alternatively, the M-complex is organized around the ERS homodimer. Accordingly, the tRip transmembrane helices α8 (green) are equidistant and similarly oriented. (**E**) Two *Toxoplasma* MSCs have been modelized to mimic *Plasmodium* Q- and M-complexes. Both models show homodimerization of *Tg*-p43 through an unknown interface (?). This homomeric interaction, if biologically relevant, would allow the formation of a unique MSC (dimer of tetramers, Figure 4C). Moreover, the transmembrane helix α8 is not predicted for *Tg*-p43.

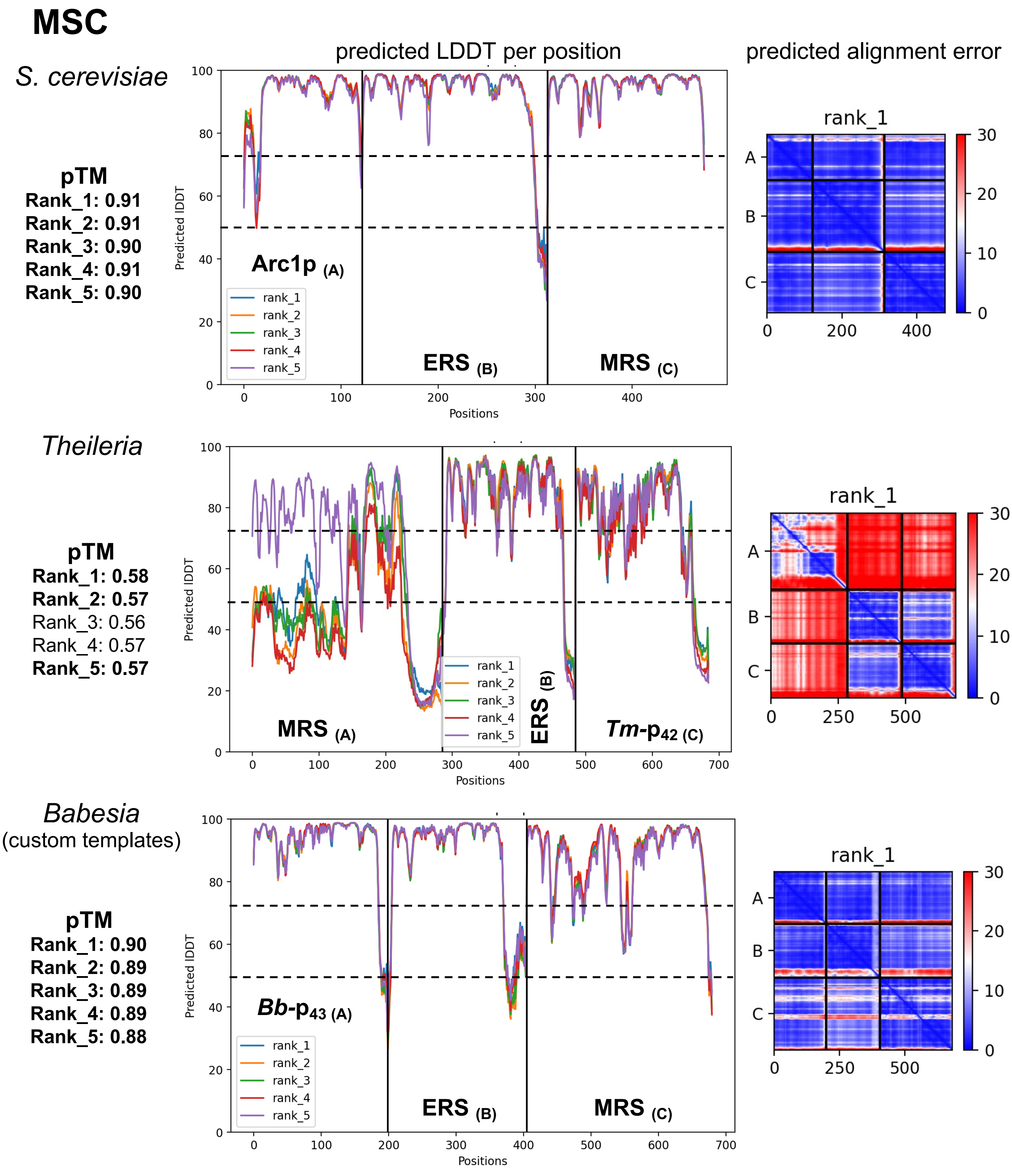

MSC
S. cerevisiae
predicted LDDT per position
predicted alignment error
pTM
Rank_1: 0.91
Rank_2: 0.91
Rank_3: 0.90
Rank_4: 0.91
Rank_5: 0.90
Arc1p (A)
ERS (B)
MRS (C)
rank_1
rank_2
rank_3
rank_4
rank_5
Predicted lDDT
Positions
rank_1
Theileria
pTM
Rank_1: 0.58
Rank_2: 0.57
Rank_3: 0.56
Rank_4: 0.57
Rank_5: 0.57
MRS (A)
ERS (B)
Tm-p42 (C)
Babesia
(custom templates)
pTM
Rank_1: 0.90
Rank_2: 0.89
Rank_3: 0.89
Rank_4: 0.89
Rank_5: 0.88
Bb-p43 (A)
ERS (B)
MRS (C)

Figure S6

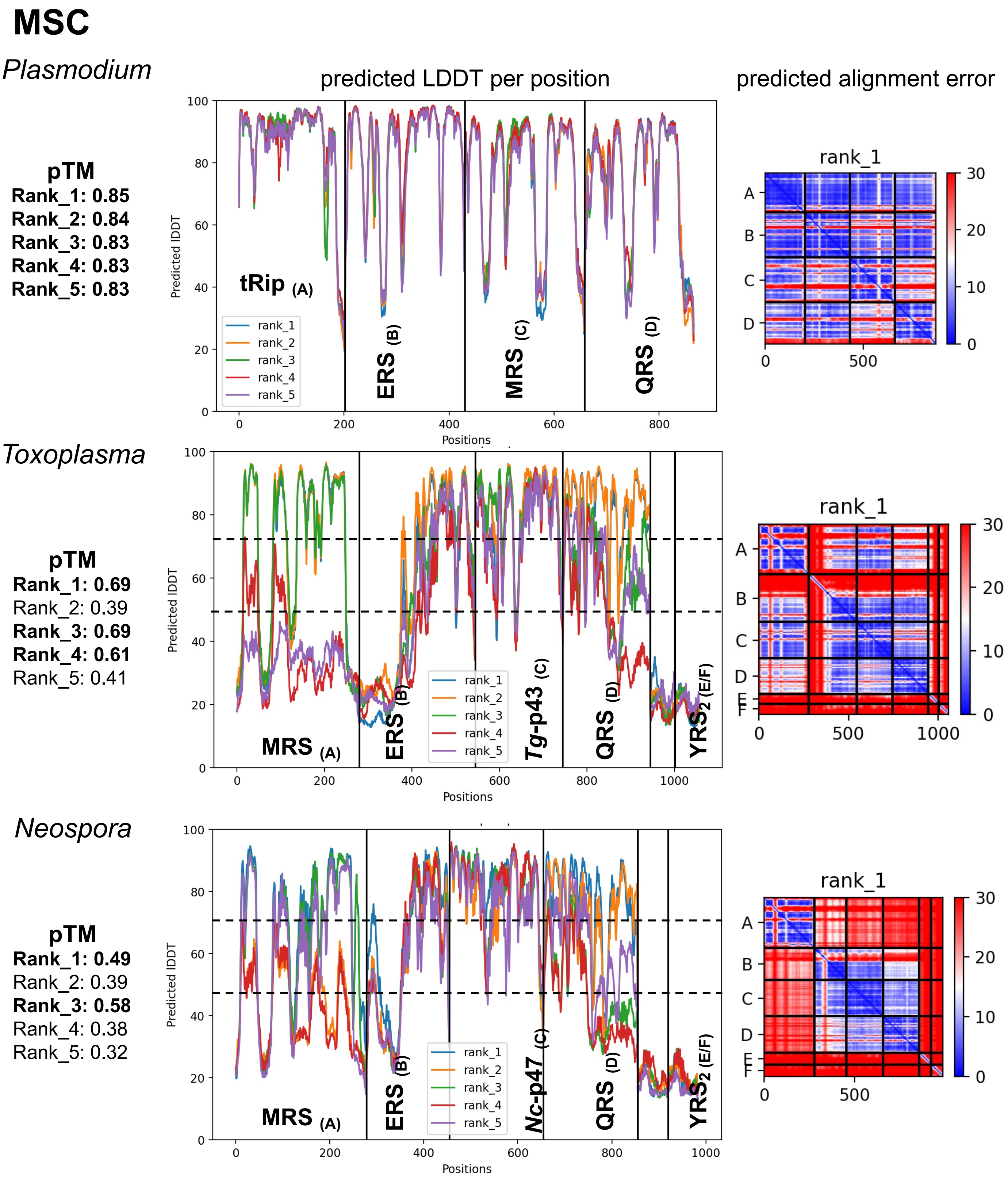

Figure S6. Confidence metrics of the ColabFold predictions for GST-like interaction network of S. cerevisiae and *Apicomplexa* parasites. See legend Figure S5

MSC

predicted LDDT per position

predicted alignment error

Plasmodium
Q-complex

pTM
Rank_1: 0.70
Rank_2: 0.66
Rank_3: 0.66
Rank_4: 0.45
Rank_5: 0.43

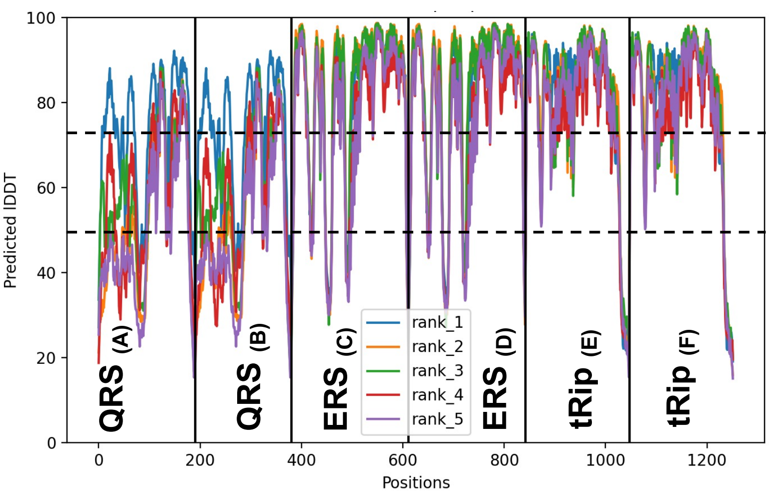

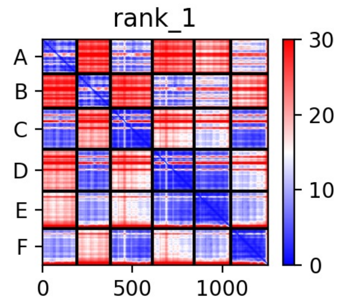

Plasmodium
M-complex

pTM
Rank_1: 0.74
Rank_2: 0.72
Rank_3: 0.45
Rank_4: 0.36
Rank_5: 0.36

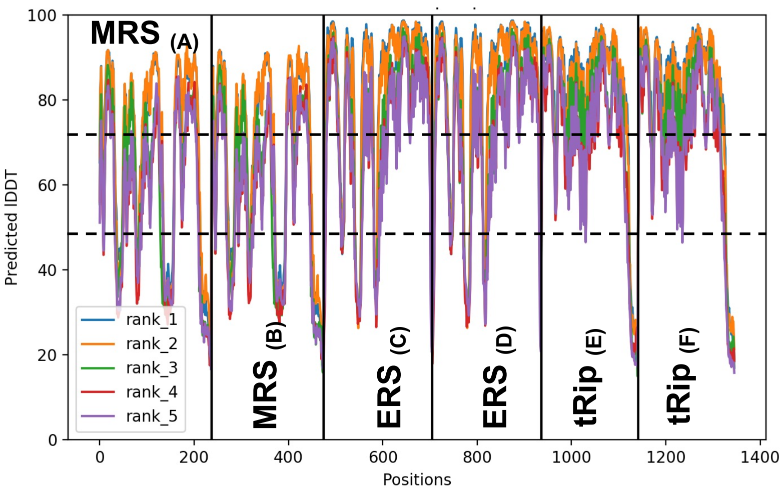

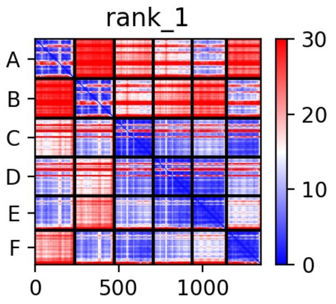

Figure S7

MSC

Toxoplasma
Q-complex

pTM
Rank_1: 0.73
Rank_2: 0.72
Rank_3: 0.66
Rank_4: 0.65
Rank_5: 0.32

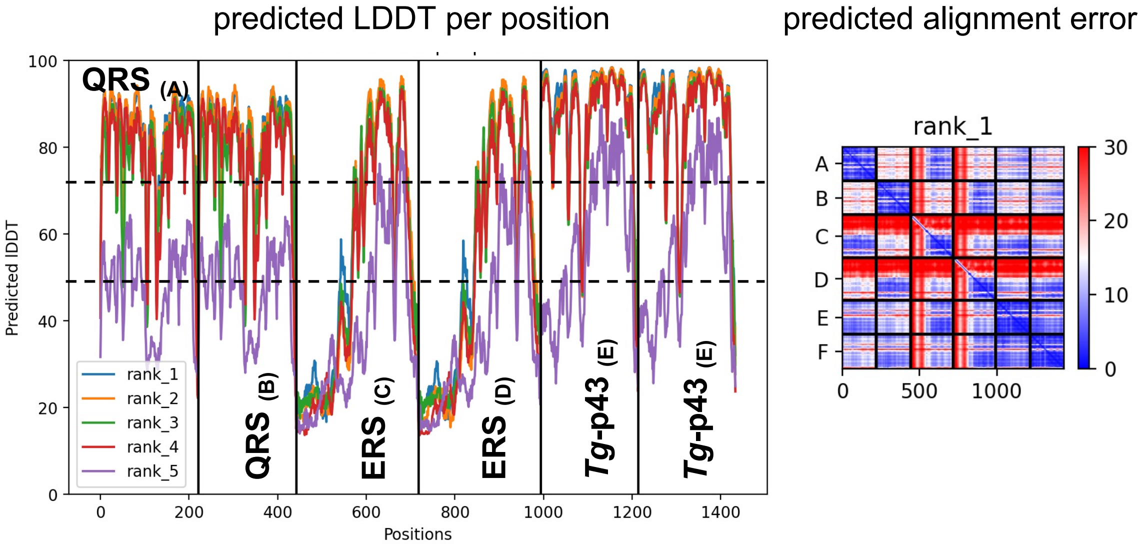

Toxoplasma
M-complex

pTM
Rank_1: 0.73
Rank_2: 0.69
Rank_3: 0.69
Rank_4: 0.56
Rank_5: 0.49

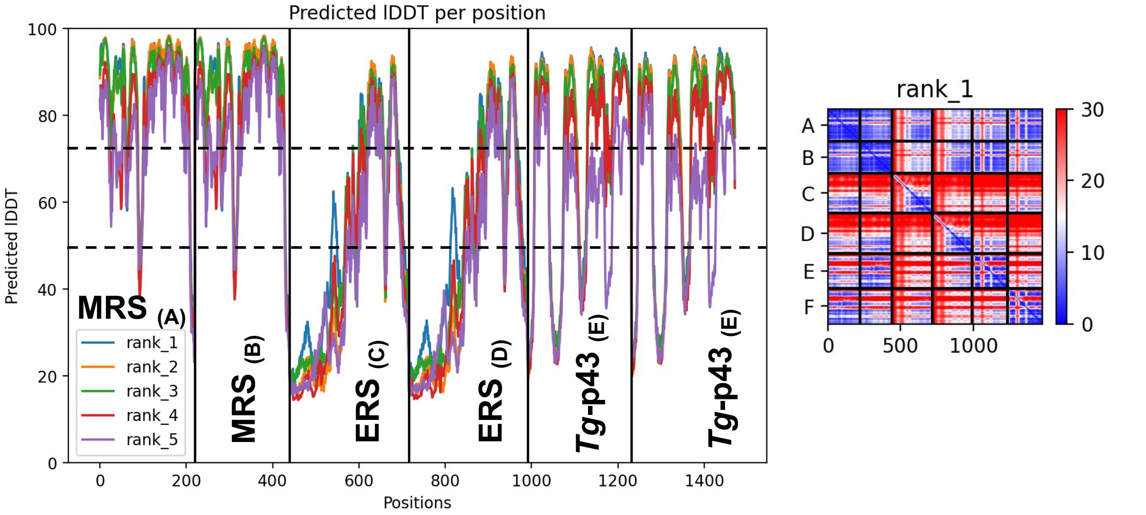

Figure S7. Confidence metrics of the ColabFold predictions for M- and Q-MSC GST-like backbones in *Plasmodium*, *Toxoplasma* and *Neosporidium*. See legend Figure S5
